# The effects of Beta-Endorphin: state change modification

**DOI:** 10.1186/2045-8118-12-3

**Published:** 2015-01-29

**Authors:** Jan G Veening, Henk P Barendregt

**Affiliations:** Department of Anatomy, Radboud University Medical Center, PO Box 9101, 6500HB Nijmegen, the Netherlands; Faculty of Science, Radboud University, Nijmegen, The Netherlands

**Keywords:** Beta-endorphin, Behavioral states, Behavioral sequences, Cerebrospinal fluid, Volume transmission, Feeding behavior, Sexual behavior, Pain, Reward, Meditation

## Abstract

Beta-endorphin (β-END) is an opioid neuropeptide which has an important role in the development of hypotheses concerning the non-synaptic or paracrine communication of brain messages. This kind of communication between neurons has been designated volume transmission (VT) to differentiate it clearly from synaptic communication. VT occurs over short as well as long distances via the extracellular space in the brain, as well as via the cerebrospinal fluid (CSF) flowing through the ventricular spaces inside the brain and the arachnoid space surrounding the central nervous system (CNS). To understand how β-END can have specific behavioral effects, we use the notion behavioral state, inspired by the concept of machine state, coming from Turing (Proc London Math Soc, Series 2,42:230-265, 1937). In section 1.4 the sequential organization of male rat behavior is explained showing that an animal is not free to switch into another state at any given moment. Funneling-constraints restrict the number of possible behavioral transitions in specific phases while at other moments in the sequence the transition to other behavioral states is almost completely open. The effects of β-END on behaviors like food intake and sexual behavior, and the mechanisms involved in reward, meditation and pain control are discussed in detail. The effects on the sequential organization of behavior and on state transitions dominate the description of these effects.

## Introduction

### States

An organism, from a single cell to *Homo sapiens*, continuously responds to input from its environment with (if all is well) an adequate action. Such input-action pairs only partially describe the organism. Indeed, the same external stimuli may give rise to a different reaction. In that case we say that the organism is in a different (internal) *state*. In this way states are defined as action tendencies. The state of an organism may vary from moment to moment. Therefore we define the state of an organism (the notion also applies to mechanical systems with sensors and actuators) at a given moment *t,* as the way it acts when receiving certain stimuli at moment *t*. Thus a state is an idealized mathematical notion, that usually cannot be known in full, but that is nevertheless very useful: it can be approximated and can serve for theoretical considerations. The notion of state is used in computer science [1] and also extensively in mathematical system theory. Prior to this, statistical mechanics described the state of a gas in a vat as elements of a space of dimension 6.10^23, where the 6 stands for the 3 position coordinates plus 3 velocity components of the 10^23 (Avogadro’s number) molecules in a given volume. Such complex states cannot be determined empirically nor theoretically, but serve to derive the well-established laws of thermodynamics.

### Approximating states, sub-states

As approximation to a state in an organism, one can consider a vector of variables having a given value [2]. Indeed, in the ideal case of having all possible values, such a vector completely determines the behavior of the organism, depending on presented stimuli. A *sub-state* is a part of this vector of values, determining only partly the input-action relation. For the notion of state one may restrict oneself to relevant forms of input and action, depending on the context of the subject matter. These are sub-states relevant in a certain scientific context. For example, in studying a species one may restrict oneself to feeding or to sexual behavior. In such cases one speaks about *behavioral states*. For *Homo sapiens* another restriction is useful: it makes sense to focus on signals available to consciousness and to intended actions. Then one speaks about *mental states*.

The notion of state is a so-called higher-order mathematical notion. A state is not involved with just one input signal and one action signal, but with a whole class of input-action signals. Making restriction eases the fact that states are higher-order mechanisms and can be helpful to obtain experimental or theoretical results. The well-known human emotions (like anger, fear, desire, surprise, disgust) are all examples of mental sub-states of a more general mental state. They are sub-states because one can be angry and fight, or angry and submissive, hence indeed some parameters are lacking.

### Phases: keeping some sub-states fixed

By the definition above, a state is momentary, occurring at a precise moment in time. A sequence of related (e.g. when a certain sub-state being fixed) states during an uninterrupted interval of time can be called a phase. An example is an animal in the state of being hungry. It is looking for food in order to eat. Usually the state of hunger remains, even if food is found and the animal is eating. In this phase of eating, the sub-state hunger is more or less fixed. It has to persist for obvious reasons: one bite of food is not enough. But the persistence of hunger is not exact; it (gradually) diminishes, for otherwise the animal would (in the condition of abundance of food) never stop eating. Another example of a phase with a constant sub-state is sleep. The mechanisms for sensorial input and muscular output are for a large part blocked. But during the phase of sleeping not all sub-states are the same: one can distinguish rapid-eye movement (REM) and non-REM sleep. In both examples phases may be divided into several sub-phases.

It should be emphasized that the notions of state, sub-state, phase, and sub-phase are all quite natural and familiar. Talking about the weather, the notion of (momentary) state corresponds to the familiar values indicating the temperature, air-pressure, direction and speed of the wind, humidity, etcetera, all at one given moment. The notion of phase that lasts for a certain time interval applies to what we usually call the weather. Indeed, a rain storm consists of an uninterrupted flow of states having in common that it is both raining and windy. Exactly how much rain is falling and whether there is also lightning (a sub-state at a given moment) may vary during the interval of the phase of the rainstorm. So there is place for sub-phases: “During the rainstorm the lightning lasted unusually long.” We see that the notion of (momentary) state is fundamental, while phase refers to a continuous time interval during which the momentary states are similar but not necessarily equal, for example by keeping some of the sub-states constant. The concept of phase is very different from that of state. There is a precise mathematical definition of what is a state. What is a phase depends on what one considers to be comparable. When exactly does a storm end? One could say: “If no longer there are gusts of wind with speed 150 km/hour.” Here there is a place for choice (possibly very relevant for aeronautics). In the case of state at a given moment, there is no choice.

### Change and maintenance of states

In an organism with a CNS, induction of a new sub-state in which a couple of parameters are to be changed, can be organized efficiently using action potentials and synaptic transmission. If, on the other hand, there is a need for maintenance of a sub-state, in the terminology above for a certain phase, there seem to be several natural possibilities for doing this. 1. Sustained neural activity, using synaptic transmission. 2. Local volume transmission (VT) to keep certain chemical parameters locally at the right level. 3. If parameters need to be changed globally into a certain direction, then volume transmission through the cerebrospinal fluid (CSF-VT) is an option (see Table 1). For animals with a developed CNS, we have argued that there is a mixed usage, in particular for neuropeptides like oxytocin and β-END [3–5]. It has been shown that the mechanisms work in parallel: a fast ephemeral axonal message transports the signal of a peptide to relevant areas; after that the slower but longer lasting volume transmission does its work. Via second messengers, the effects of VT may last much longer, up to months, years or even life-long [6–11]. This may be related to the question why there are so many neuropeptides and what is their relation to the variety of different behavioral states.

**Table 1:** **The effects of β-END in the CNS**

β-END effects in the CNS	Short term	Long term
**Local**	Synaptic transmission	Volume transmission
**Regional and global**	Volume transmission	Volume transmission via CSF

In the present as well as in our preceding paper (Veening et al. [5]) a variety of CNS-effects have been described for β-END. The available evidence suggests that this neuropeptide exploits all kinds of messaging available in the CNS.

Numerous findings in the literature can be explained by simply assuming that neuroactive substances, released at specific points in the brain along the ventricular system, reach their distant target areas by ‘going with the flow’ of the CSF. This particular kind of long-distance-VT has been included in all discussions concerning VT [6, 11–15] and in fact β-END was among the very first substances mentioned in relation to VT [9, 11]. Meanwhile, the effects of substances released from particular parts of the brain into the CSF to target distant brain areas have been studied for a variety of substances such as vasopressin, corticotropin-releasing-factor, gonadotropin-releasing-hormone, melatonin and oxytocin [3, 4, 16–22]. Recently, we have reviewed the available evidence showing that β-END is another neuropeptide that can be released into the CSF to affect distant brain areas [5]. Most neurons producing β-END are located in the arcuate nucleus (ARH) of the basal hypothalamus, alongside the third ventricle, but an additional, smaller group has been observed in the caudal brainstem. The involved ARH-neurons produce a mixture of neuropeptides and are known as proopiomelanocortin- (POMC-) neurons (for further details, see [5]).

The present review discusses more specifically the potential of CSF-flow to influence a number of brain areas together, to induce behavioral (state-) changes. The behavioral data, provided below, show on the one hand that certain brain manipulations with β-END have a special effect on specific behavioral transitions, thereby effectively blocking transitions to another behavioral phase. On the other hand, β-END induces general behavioral effects, described by several authors as ‘a state of well-being’ (see below), which also clearly suggests that the effects of this neuropeptide are state-related. The present review focuses on such behavioral effects of β-END and in addition we discuss states and their selection and preservation on the basis of the Turing-model [23] inspired by Turing [1]. Actually it is a hybrid model that is also inspired by the neural nets introduced in Turing (1948), see [24, 25] describing artificial neural nets, a connection already pointed out previously [8].

### The sequential nature of behavioral states in human cognition

In Zylberberg *et al*. [26] and Barendregt & Raffone [23] human cognition has been independently described as a 'discrete hybrid Turing machine’. This means the following: 1. [Discreteness] actions proceed in a serial way (one after the other, like a fast ticking clock, not continuously); 2. [Turing machine] these actions do not only depend on the stimulus (a demonstrable drawback of behaviorism), but also on the state of the organism/machine; 3. [Hybrid machine] how a given stimulus and state produce an action is determined by a parallel neural net (unlike in the classical Turing machine, where these transitions are described by an explicit table). The model is quite simple: important events, state changes and actions, are occurring in a discrete serial fashion, depending on the previous state and the input. Among the possible actions, focusing attention is an important one. This is the full model description. The discreteness of human cognition is supported by psychophysical evidence on visual illusions and periodicities in reaction time (for a review see [27], as well as by electroencephalographic evidence about discrete brain microstates, (see [28]. Paper [27] emphasizes different aspects than does [28]. For example the first paper beautifully answers the question of John von Neumann how it is possible that human cognition answers questions with high accuracy while there is biological noise. The answer by Zylberberg *et al*. [26] is convincing: “by discretization” (like a CD avoids the noise of an old-fashioned vinyl record). The second paper [28] emphasizes the use of states, and employs the notion of a universal Turing machine (programmable computer) in which states can be used as input. This enables modification of automatic behavior.

### The sequential organization of animal behavior: a ‘funnel model’

**Figure 1:**
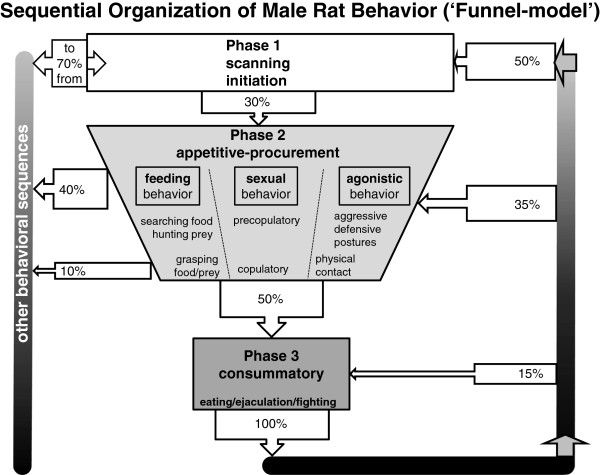
**The Funnel-Model of the sequential organization of the male rat behavior.** The model is based on a transition analysis of the behavioral elements of the male rat occurring during a series of experiments, which included feeding behavior, sexual behavior and territorial aggression, combined with the effects of electrical stimulation of the ventromedial hypothalamic nucleus (VMH). Explanation: see text. We have coined this model the ‘Funnel-Model’, because it illustrates clearly that in phase 1 of the behavioral sequence the animal is relatively free to make choices leading to any possible ‘consummatory act’, or in a wider view, to any behavioral state. Phase 1 can be characterized as a transitional situation, from where any behavioral sequence leading to a specific consummatory act can be performed or from where any possible behavioral state can be reached. At the end of phase 2 the situation is completely different: the male is ‘bound to’ perform the consummatory act, (mostly consisting of a series of physiological reflexes) and the opportunity to select other behavioral transitions is temporarily blocked. Only after completing the consummatory behavior, the ‘freedom’ of phase 1 is available again. The ‘Funnel-model’ illustrates on the one hand that physiological and brain mechanisms are working to support behavioral perseverance and to keep behavior directed to a specific goal, while on the other hand, especially in phase 1, the opportunity is raised to choose another strategy, or to pursue another goal or to reach another state.

In a behavioral study of rats [29, 30] the structure of feeding, sexual and agonistic behavior was analyzed by means of an extensive transition analysis of the successive behavioral elements. In some experimental situations, regular transitions were interrupted by electrical stimulation of the ventromedial hypothalamic nucleus of the freely moving rat. This approach showed not only the normal succession of behavioral elements in each of the behavioral sequences, but also the interruptive effects of the medial hypothalamic stimulation. These effects were strongly dependent on the moment of delivery during the behavioral sequence: during the ‘scanning’- or initial phase the normally succeeding transitions towards the appetitive and consummator phases appeared to be completely blocked. When stimulation started during the succeeding, appetitive, phase, the experimental animal tried to ‘switch back’ to the initial phase, as long as possible. However, when the brain stimulation was delivered in the final phase, animals tried to ‘complete the sequence’ before returning into the initial phase. These findings together are the basis for the ‘Funnel Model’, [29–31] describing the organization of behavior of the male rat, as depicted in Figure 1.

In phase 1 the resident animal is just scanning the environment, with some sniffing and locomotion, and for the observer it is not yet clear in which direction behavior will develop. If, however, palatable food or an estrous female or a male intruder is introduced into the resident’s cage, its behavior rapidly changes into the appetitive/procurement phase, with characteristic and species-specific and goal-directed behavioral elements to obtain the food/prey, to perform (pre)copulatory activities or to approach and threaten the male intruder. In the final consummatory/executive phase, food consumption, or ejaculation or biting and fighting occur, until the intruder shows submission or leaves the field. In this final phase, the behavior contains many ‘reflexes’, organized at the level of the brainstem and spinal cord, like chewing and swallowing, ejaculation as controlled by the spinal ejaculation center [31–34] or an extremely fast series of biting and fighting movements. At the end of the consummatory phase the animal may return to the initial phase, when (temporarily) satiated by food or ejaculatory activities, but it may also enter the second phase immediately again if satiation (= ‘negative feedback’-signals) did not occur sufficiently.

In Figure 1 the arrows indicate the number of transitions from and to the successive behavioral phases. At the left side, it is shown that in the early initiation-phase behavioral transitions occur frequently (70%) towards other behavioral sequences. Early in phase 2 a considerable number of transitions (about 40%) still occurs in a direction not leading to the expected consummatory phase, but later in the appetitive phase such an exit-choice is made in less than 10% of the transitions. For that reason we have coined the term Funnel-Model to describe the sequential organization of behavior, because it suggests that the possibility to choose for another behavioral goal becomes more and more restricted. In the laboratory situations studied, 50% of the animals, entering Phase 2, continued towards the appropriate consummatory elements, apparently after some point of no return. The animal had no choice other than completing the sequence and only after completing the total sequence, the animal could either repeat a part of Phase 3 (15%), or enter Phase 2 again (35%) or, under our experimental conditions, enter Phase 1 again (50%). We consider an animal behaving in Phase 1 as least constrained, meaning that all behavioral options are open depending on the internal state and the external stimuli, and that an animal at the very end of Phase 2 is most strongly constrained, and virtually bound to finish the complete sequence by performing the mostly reflexive acts composing the consummatory phase 3.

This Funnel-concept was strongly supported by the, mostly disturbing, effects of electrical stimulation of the ventromedial hypothalamic nucleus (VMH). During the 30-sec stimulation periods, animals did not or hardly entered phase 2. When, however, stimulation happened to start at the very end of Phase 2, animals seemed to finish the sequence as quickly as possible. During VMH-stimulation at the end of the sequence, animals never returned to phases 2 or 3, but immediately entered a vigilant version of Phase 1, remaining there during the 30-second intermittent stimulation period [29–31]. The Funnel-shape of the behavioral progression during a sequence plays an important role in a wider context of animal behavior: coping mechanisms and behavioral states. Considering feeding, sexual behavior and agonistic activities as just three of a variety of possible behavioral sequences, we wish to address two aspects specifically: on the one hand, goal-directed behavior asks for perseverance for a specific goal, and the performing animal should not be diverted by irrelevant external or internal factors for a while. Behavioral funneling supports the animal to stay in a given behavioral sequence or behavioral state, to stay on track until the goal is reached. On the other hand, less constrained periods, like Phase 1 in between specific behavioral sequences, are necessary as switching points in order to allow the animal to choose another behavioral strategy, for entering other states and/or to pursue other goals. The funnel-shape reflects these opposing characteristics in a continuous flow of behavioral performances. β-END appears to be involved in this behavioral flow and the switching points (see sections 3.1 and especially 3.2).

### Towards a general role of β-endorphin

Over the last decades, numerous reviews have appeared on the behavioral and physiological aspects of β-END [35–63]. From these, it becomes clear that β-END is involved in a wide variety of functions ranging from the cellular to the behavioral level. Many reviewers discussed the placebo effects possibly occurring on the administration of β-END and other substances influencing the μ-receptor [64–70]. Several of the mentioned reviewers of the effects of β-END proposed unifying concepts to embrace this variety into a general behavioral function, but their proposals seem to be pointing in different directions.

In 1982 Henry assumed that all general effects, for example those affecting the pain-regulating systems in the spinal cord, were induced by circulating opioids [71]. The paper did not yet consider the possibility of β-END messages via the CSF. Henry asked, however, special attention for the possibility that “the activation of a number of functions together may be due to a global activation of opiate receptors throughout the CNS”, which is consistent with the main thesis of our present paper. In the summary (p 239), after comparing the effects of stress, and sexual activities with vigorous dancing and states of trance, Henry concludes that mild activation of the β-END system induces a state of well-being, while stronger activation results in analgesia and euphoria. On the other hand, “when the endorphin system is hypoactive, ……, an increased drive ensues to satisfy a deprived state, whether this is an appetite for food, water, social contact, or sexual satisfaction, etc.” [71]. This paper contained the clear hypothesis that the effects of endorphin depend on the activity state of the endorphin system. In 1984, Akil *et al*. concluded that “The multiplicity we behold in studying endogenous opioid function is dizzying”, [72], focusing in their review mainly on stress, analgesia and cardiovascular control mechanisms. In 1985 the role of β-END in learning and memory processes was addressed [73–75]. Izquierdo and Netto showed that “ a variety of behavioral experiences activate the β-END system, apparently as a result of novelty; that this activation is mediated by the septo-hippocampo-subicular system; and that this seems to play a role in regulation of the retrieval of learned behavior …….” [74]. Due to its rather long recovery time, the arousal of the β-END system “must be reserved for events that are particularly striking to the animal”. “β-END may obviously play a very important role in adaptive behavior” as well as in developing (alternative) coping strategies (ibidem).

In 1986 the μ-receptor system was discussed [59]. After referring to a variety of behavioral and physiological effects, as described in the literature, Panksepp continues as follows: “Although many of the behavioral effects could be subsumed by the principle that opioids elaborate pleasure or habit processes in the brain, such a perspective would not explain the peripheral physiological effects of opioids. Suppose we broaden the scheme and postulate that the global function of opioid systems (μ and perhaps δ) is to counteract the influence of stress. Although stress is a construct beset by serious operational and conceptual difficulties*,* if we consider any major perturbation of physiological homeostasis to be a stress, with opioid arousal being a cardinal counteracting influence, most effects reported for the opioid system fall into place” [59]. “The desirable affective effects of opioids can be understood as the psychic component of a brain process that helps return activity in perturbed neural circuits back to normal”. In addition, “there is an arousal component to opioid action in the brain, especially on cells of the mesolimbic DA (dopamine) pathways, which appears to elaborate the euphoric effects of opioids”, possibly to “promote homeostasis-sustaining behaviors”. “Pleasurable opioid arousal invigorates those active post-homeostatic behavior patterns such as rough-and-tumble play, that are expressed fully only when other bodily needs have been fulfilled” [59]. After some final remarks concerning stress-induced-analgesia, Panksepp concludes that “Perhaps stress-induced-analgesia would be more properly called relief-correlated-analgesia”.

Some years later, Herbert summarized the functional aspects of β-END as follows: this peptide “is particularly concerned with regulating reproductive physiology and is part of the mechanism whereby reproduction is controlled by (and thus responsive to) various elements in the external environment, including social and physical stress” [38]. Central β-END is released during various forms of stress and “is a prime example of a peptide whose principal function seems to be *to inhibit responses in conditions under which they would be disadvantageous”* (p 739; emphasis is ours). “β-END-containing systems can be accessed by changes in either the physical or social environment that signal adverse conditions for reproduction. The result of activation of this system is a common one: suppression of reproduction” [38].

At first sight, the comments of these successive reviewers may seem difficult to reconcile. However, taking the idea of a main role for β-END in creating a general state of well-being and pleasure (up to euphoria and trance) as a starting point, the differences between the reviewers tend to disappear. This state can be contrasted with alternative states like stress and a variety of motivational states. As proposed by Panksepp, a variety of stressors may induce perturbed neural circuits, and changes towards an opposite preferred endorphin state can be described, supported by activation of the mesolimbic dopaminergic pathways, creating reward when approaching the desired state. Basically, this is not much different, however, from what happens when an animal returns from a specific motivational state (hunger, thirst or sexual arousal) to a state of satiety and homeostatic balance, after performing the appropriate behavioral sequence [30, 31, 38]. Such motivational states may be the result of serious homeostatic needs, which can be very stressful. Concerning food intake, this may imply that eating may be increased or decreased to reach the preferred weight level. We suggest that β-END influences brain-activity towards a state of balance and well-being. In order to keep the brain in this preferred state, β-END apparently allows the animal to perform feeding and drinking activities (see below) as well as social grooming [76], which relieve stress and may restore a disturbed balance. Breeding activities, however, under non-optimal conditions, that would seriously disturb the state of well-being, are apparently inhibited [38, 77, 78], just like painful stimuli that have to be avoided [71, 72, 79]

Our conclusion concerning the general role of β-END is therefore the following. On the one hand, β-END may allow and stimulate behaviors that normally restore a state of homeostatic balance and well-being, and on the other hand it may inhibit behavioral changes that potentially disturb this preferred state. On the basis of this statement, several interesting and specific questions can be raised about the behavioral effects of β-END, about specific behavioral transitions, (as studied by Herbert *et al.*) [71, 72, 79–85], as well as the effects of meditation. These will be discussed in the next sections.

## Behavioral regulation by β-Endorphin

### Regulation of food intake

The POMC neurons in the ARH are strongly involved in the regulation of food intake, at the sensory side equipped with specific receptors and at the effector- side with mechanisms controlling food intake. Most of the POMC-neurons express leptin receptors [86, 87], while processing of the POMC-derived peptides is regulated by energy balance [87–90]. On the effector side, it is fully clear that hypothalamic POMC-neurons play an important role in the regulation of food intake [86, 90–97]. Among the POMC-derivatives, alpha-melanocyte-stimulating hormone (α-MSH) appears to be the main one, exerting an inhibitory effect on food intake as shown in human as well as animal research, via the melanocortin receptor types MC3 and MC4 [90, 92, 94, 95, 98–103]. The role of β-END in food intake regulation appears to be less prominent and more modulatory in character. From the earliest studies on, it became clear that central administration of opiates, among them β-END, had a stimulating effect on food intake [105–112]. The stimulating effects of other neuropeptides, like galanin (GAL), were also mediated by β-END release [113] while on the other hand serotonin seems to play a role in the feeding effects of β-END [114–116]. Several recent findings have complicated the role of β-END in food intake. Its levels in both CSF and plasma were elevated after intake of palatable sucrose solutions in rats [117], while ingestion of a fatty meal induced neuronal activity in the β-END neurons, apparently after oropharyngeal stimuli arriving via the glossopharyngeal nerve [118]. This finding is in agreement with an earlier observation showing that, in the rat, β-END plays a special role in the hedonic preferences for dietary fat [119]. In addition, β-END deficient mice are less willing to work for palatable food, suggesting motivational changes in the appetitive phase [120].

Maybe, the short-term effects of β-END on food intake are different from the long-term effects, since intracerebroventricular (icv) infusion of β-END in rats stimulated food intake but chronic infusion did not sustain these stimulating effects [104]. Recently it was also observed in transgenic mice, that that short- and long term effects on POMC-neurons may be different. The long-term effects seem to be more complementary to the general POMC-effects [120, 121]. After a temporary initial increase, male rats showed a lower level of food intake after a few days of chronic icv β-END administration [122]. This weakly inhibitory effect is in agreement with results obtained from β-END-KO mice, which gained an additional 10 – 15% of body weight compared to wild-type controls. These KO mice showed an increase in food intake without changes in basal metabolic rate. The increased body weight consisted completely of an increased amount of white body fat and was only observed in male mice, not in females [121]. Finally, it has been suggested already in the early nineties [123–126] that the role of β-END in food intake is mainly sustaining, instead of playing an initiating role.

The question that has to be raised now is: How far it is possible to integrate this variety of findings into a general role for β-END in the regulation of food intake? The short-term effects of β-END seem to be most prominent and decrease the appetite-inhibiting effects of α-MSH [104]or a preceding stressor [102] and thereby β-END may play a special role in the appetitive phase of feeding behavior. These effects can be linked to the reward system which induces positive feedback stimuli in the appetitive phase [127–129] to sustain food intake of palatable food [119, 121, 126]. Apparently, “β-END selectively affects a motivational component of reward behavior under non-deprived conditions” and “in the appetitive phase, β-END release increases the incentive value of food as a primary reinforcer” [120]. These behavioral effects certainly contribute to restore a state of well-being, as postulated for a general role of β-END. The question as to how far the long-term effects of β-END, consisting of moderate inhibitory effects on food intake, contribute to long term body homeostasis and weight regulation (only in males), or whether these have to be considered merely as a side-effect of β-END supporting some other POMC-derived peptide(s), remains open for further research. In conclusion, the central effects of β-END on food intake are modulatory and play a main role in the introductory/appetitive, goal-directed phase of feeding behavior. In this phase behavior can be most easily adapted to obtain or preserve a preferred state [30, 31] or to choose an alternative coping strategy.

### Sexual behavior

Despite the fact that the effects of opioid administration have frequently been described as reaching an orgasmic state of euphoria, the effects on sexual behavior are generally inhibitory [130]. The acute and long-term effects turned out to be complex [131–133], including those of β-END, which plays a role in male as well as in female sexual behavior. In the male, β-END is involved in the regulatory control of testosterone, via luteinizing hormone (LH) and gonadotropin-releasing hormone (GnRH) mechanisms. The effects are mainly inhibitory [132, 134–137]. The details of the observed effects were, however, complex [131, 133, 138], rather variable, for instance those on ejaculation latencies and of naloxone [134, 139–142]. Also the effects of ejaculation, and erotic stimuli (in humans) did not lead to consistent results when measuring the peripheral β-END levels [132, 143–147].

Similar to what we have discussed for the control of feeding behavior, several findings suggest that β-END plays an important role only in specific phases of sexual behavior, especially the precopulatory, appetitive phase. In 1981 Meyerson observed that after icv administration amicable contacts between animals increased whereas sexual responses were decreased [148]. Later studies supported a specific role of the μ-receptors in pair-bonding [149]. Numerous studies have reported the deteriorating effects of stressors on male sexual performance [150–155]. In addition, the analgesic effect of copulatory activities has been noted repeatedly [147, 156, 157]. All findings, taken together, strongly suggest that the main role of β-END is played in the appetitive, precopulatory phase of sexual behavior, to pave the way for the copulatory activities themselves. In this phase, the dopaminergic system is also involved, providing a rewarding basis for the ongoing activities. The effects of β-END during the appetitive arousal state are related to its stimulating effects on the reward systems and an important role, especially in the medial preoptic area (MPOA) [130, 131, 133, 158–172]. In 2004, it was shown that sexual behavior and especially sex-associated environmental cues activate the mesolimbic system in male rats. This activation induces internalization of μ-receptors in the MPOA within 30 minutes after mating and this internalization was still evident about 6 hours later [168, 173]. Naloxone prevented this internalization but not the concurrent Fos- expression of the MPOA-neurons [173]. In 2007, it was shown convincingly that POMC-neurons in rats were activated by the arousal aspects and not by sexual activities themselves [167].

These findings are in full agreement with earlier suggestions concerning the effects of β-END on interpretation and impact of environmental stimuli [174, 175]. Moreover, these findings suggest that the neural mechanisms involved in either the arousal/precopulatory phase or the copulatory phase of male sexual behavior show some fundamental differences. In the rat, an initial appetitive, precopulatory phase of approaching and investigating the female, is usually followed by a sequence of copulatory activities (mounts and intromissions) eventually leading to an ejaculation [176]. After a post-ejaculatory interval of several minutes, the whole behavioral sequence may start again. (See Figure 1 for an overview of the behavioral sequence.) β-END seems to play its specific role especially in the appetitive arousal phase (and in the post-ejaculatory period), but not during the copulatory activities themselves.

Support for the idea of ‘phase-specific’ effects of β-END was obtained by the group of Herbert [38]. They injected β-END bilaterally by micro infusion via brain cannulas into several specific rat brain areas and made some striking observations. After showing the generally inhibiting effects of β-END and the involvement of different brain areas [80–82], it was observed that the inhibitory mechanisms of the MPOA and the medial amygdala (MeA) were very different, but had eventually the same effect. In both brain areas a specific behavioral transition in the usual sequence of (pre)copulatory events turned out to be of crucial importance: the transition between the appetitive, precopulatory, phase and the copulatory phase turned out to be important for the β-END control. After β-END administration into the MPOA, investigative activities were normal but the males never entered the copulatory phase, unless the β-END administration started after the initiation of the copulatory activities. In that case, these were performed as usual. When, however, a new female was introduced, the same precopulatory-copulatory transition was blocked again [38, 85]. On the other hand, the same behavioral transition turned out to play a similar important role in the MeA, but in a completely different way. Now the precopulatory phase was completely suppressed by the β-END administration and the animals never made the transition to the copulatory phase. However, copulatory activities themselves were completely normal, as observed when β-END was administered in the later copulatory phase [38, 83, 84]. These transition-effects were not caused by sensory (olfactory or visual) disturbances possibly induced by the β-END infusions [38].

Apparently, the transition step between precopulatory and copulatory phases of masculine sexual behavior is under β-END control and copulation can be effectively blocked by either prevention or an endless continuation of investigative activities. Since the MeA, especially its posterodorsal part, receives genitosensory as well as olfactory information, and since it is reciprocally connected to the MPOA [31, 32, 177–185], this part of the neural circuitry not only plays a role in the induction of the post-ejaculatory interval, but may just as well be involved in the general control of ejaculatory activities. Manipulation of a specific but crucial behavioral transition is an extremely efficient way to induce or block the occurrence of specific parts of behavioral sequences! Herbert concluded from these and other studies that β-END “is a prime example of a peptide whose principal function seems to be to inhibit responses in conditions under which they would be disadvantageous” [38] (p 739). In our view, β-END appears to play a dual modulatory role in male sexual behavior. On the one hand it facilitates the appetitive phase by reducing stress and potential pain and by activating the reward system [130, 131, 133, 156, 165], on the other hand by inhibitory effects on specific transitions in the regular copulatory pattern in addition to short-term- (post-ejaculatory refractory period) or long-term- (stress, seasonal effects) suppression of reproductive activities. Since the copulatory phase itself is hardly influenced by β-END, these behavioral effects fit a more general anti-stress-function: facilitating behavior towards a state of well-being, reward and even euphoria but inhibiting behavior under inappropriate, potentially stressful, conditions. This dual role of β-END on male sexual behavior is rather similar to what we concluded about its role in food intake.

In the female, β-END mainly has an inhibitory effect on receptivity, lordosis behavior and reproduction [40, 132, 186–193]. This inhibition occurs via the GnRH system in the medial preoptic area, which receives numerous β-END contacts [137, 193–211], as well as via additional μ-receptive MPOA neurons [137, 168, 212–217]. Obviously, the inhibitory effects are estrogen-dependent [168, 193, 218–222], but additional neuroactive substances are also involved in its regulatory control, like neuropeptide Y (NPY), GAL, serotonin (5-HT) and GABA [168, 195, 223, 224]. Interestingly, however, icv-studies showed that β-END could have also a facilitating effect on lordosis, given the proper conditions and location. In ovariectomized rats, primed with estrogen (and progesterone) lordosis in response to male mounts was only inhibited via high-affinity μ-receptors, but facilitated via low affinity δ-receptors [132, 186]. The facilitating effects of β-END were restricted to the first 6 hours after estrogen administration [193–199]. This facilitatory effect changed into an inhibitory effect over the next 6 hours of estradiol-benzoate (EB)-priming [193, 196–199, 225]. The mechanisms involved were determined to be not only time-dependent but also location-dependent. If crystalline EB was implanted in the septal-preoptic regions, β-END effects were facilitatory, if implanted into the ventromedial hypothalamus the EB-implant had an inhibitory effect, while at the level of the mesencephalic reticular formation no effects were observed [225]. In 1997, Gorzalka and coworkers showed that the effects of β-END administration on lordosis were ventricle-dependent: in the lateral ventricle it worked facilitating, but in the 3rd ventricle it had an inhibitory effect, probably due to the activation of different populations of opioid receptors [226]. This observation is reminiscent of other location-dependent effects, as observed by [225] but Gorzalka *et al*. also noted that β-END had no effect on proceptive behavior, like ear-wiggling [226]. This observation is especially interesting, because it suggests again a phase-specific effect: that β-END may have differential effects on the early introductory/arousal phases of female sexual activities compared to the succeeding copulatory phase. These differential effects are reminiscent of our earlier discussion for male sexual activities, but it is obvious that additional information is needed. In conclusion, the effects of β-END on female sexual behavior appear to be modulatory, as in the male. Depending on the brain areas affected and the state of the opioid receptors involved as well as the gonadal state of the animal, β-END may have facilitating or inhibiting effects on lordotic behavior. In how far these effects contribute to a state of well-being, as suggested for the male sexual activities, deserves further experimental attention, but the avoidance of inappropriate breeding conditions, as hypothesized by Herbert [38] certainly contributes to this state.

### Reward and meditation

β-END is known to induce euphoria and to have rewarding and reinforcing properties [71, 227, 228]. Numerous recent reviews discussed the involvement of mu-receptors in the liking and wanting aspects of food reward as well their role in a variety of eating disorders [229–246]. Concerning the rewarding aspects of sexual behavior and the involvement of opioids, a similar series of papers and reviews is available to support this functional relationship [166, 167, 247–254]. The bidirectional interactions between the opioid systems, including β-END, and the mesolimbic (and incerto-hypothalamic) dopaminergic systems compose the neural substrate for the rewarding effects of eating and sexual behavior. These interactions can be considered as crucial components of the mechanisms involved in motivational drives and goal-directed behavior. The motivational effects of numerous neuroactive substances are mere reflections of their inhibitory or excitatory actions on this dopaminergic reward system, extending between the ventral tegmental area (VTA), the nucleus accumbens and the MPOA. “The (induction of a) reward state in males and females is mediated by opioids and the medial preoptic area of the anterior hypothalamus is a crucial site for sexual reward” [249, 255].

In addition to the natural rewards obtained by specific behavioral actions, numerous drugs are able to influence this reward-system directly or indirectly without any specific behavioral activity. These substances induce drug-seeking behavior and addiction with all of their deleterious consequences for the individual and society [227, 228, 240, 256–259]. β-END plays an eminent role in addiction because of its mutual modulatory relationships with the mesolimbic dopaminergic system [256, 268, 260–267]. Its rewarding role in cocaine, alcohol and nicotine addiction is fully supported by the presently available evidence, while the evidence for a role in addiction of tetrahydrocannabinol (THC), the psychoactive component of marijuana, seems to be more circumstantial as yet [228, 257, 258, 261, 268]. Concerning addiction-related stress control, β-END seems to play a prominent but complicated role in the successive phases involved in addiction [228]. While enhancing the rewarding properties of the addiction-related behaviors, β-END diminishes the activity of the stress-related circuitry, (involving the locus coeruleus and the CRH-neurons in the paraventricular hypothalamic nucleus), induced by the anxiogenic side-effects of cocaine [269–273] or by unpredictable distress [273, 274]. The extinction-phase, as a stressful transition between the maintenance- and the withdrawal phases [228], induces again a tremendous increase in β-END release in the nucleus accumbens [276]. During the withdrawal phase, drug desire (craving) remains high while levels of β-END are steadily decreasing [228, 268, 277–279].

Generally speaking, the role of β-END in reward and addiction can be described as follows: on the one hand it may enhance the initial rewarding properties of the (new) behavior or drug, while on the other hand it softens the stressing side-effects of the drug use or other aspects of the addiction-related behavior. Maybe these roles can be considered as the two sides of the same coin, because of the mutually-inhibiting effects of the reward circuitry versus the stress circuitry [280]. However, several findings suggest that the relationship between stress and reward is more complicated than a matter of mutually inhibitory circuitry. In fact, the relationship shows a remarkable similarity to the biochemical aspects of the adrenal stress response, where adrenalin serves the function of making all bodily reserves available for handling the challenge, while at almost the same moment the corticosteroids start their, partially counteracting, anabolic activities for restoring the homeostatic balance in order to be able to cope with future challenges [281, 282]. We assume that a similar balanced relationship is also effective with β-END if processes related to addiction activate the stress-circuitry.

Generally, the neuronal circuitry activated by stressors involves such brain areas (or parts of) as the nucleus of the solitary tract, the parabrachial nuclei, the locus coeruleus, the central amygdaloid nucleus, the bed nucleus of the stria terminalis, the hypothalamic paraventricular nucleus, the hippocampal formation and cortical areas such as the insular and anterior cingulate regions. Depending on the nature of the stressor, physical or psychogenic, the brain areas mentioned may play a more or a less prominent role in activating the neurons of the pituitary-adrenal axis via the corticotropin-releasing-hormone (CRH-) neurons in the paraventricular nucleus of the hypothalamus [283–285]. For example, in the case of physical illness, the lower brainstem areas may play a more prominent role than during chronic, psychogenic stress [286–290]. To complicate matters even one step further: substances like ethanol also play a role in these interactions, as they have been shown to influence anxious behavior, behavioral despair and the effects of stressors on the tail-suspension test or on novelty-suppressed feeding in mice [102, 281, 291]. These interaction-effects are too complicated to discuss them further here, but they show convincingly that β-END is directly or indirectly involved in a range of behavioral effects (feeding behavior) and physiological effects (adrenal size).

Apparently, a CRH-dominated stress circuitry is activated under various circumstances. While it has been observed that CRH in the CSF regulates the expression of the μ opioid receptor [292], the nociception related endothelin system is regulated by β-END levels in the CSF [293]. Interestingly for the present review, however, are the numerous observations showing that activation of the CRH-neurons induces almost immediate activation of β-END neurons, inhibiting further release of CRH [268, 269, 271–273, 294–319]. This suggests that, similar to the ameliorating effects of the corticosteroids on the release of adrenalin, β-END is invoked immediately to modulate the maladaptive effects of the neural stress response. Activation of a rewarding feel-better-circuitry would be most effective to counterbalance stressor-effects, not only for suppressing pain (see below) if necessary to escape a predator, but also to cope with minor daily and repetitive challenges. Apparently, the neural circuitry involved is a complex mix of neuronal and CSF-signals.

Meditation has been shown to be very effective to counterbalance stress effects throughout the ages and its effects clearly stimulate the levels of β-END [296, 297, 307]. The central CSF-levels have not been measured yet under these circumstances. In a recent paper, however, studying ecstatic meditation using functional magnetic resonance imaging (MRI) and electroencephalographic techniques, it was observed that not only superficial cortical brain areas are involved in the effects, but that activation of the reward-system (the dopaminergic fibers contacting the accumbens nucleus) also occurs [294]. This suggests strongly that central parts of the CNS are involved in the effects of meditation, but additional supporting evidence is certainly needed. In the last 2 years, more than 100 papers have appeared about the effects of mindfulness-based stress reduction, that is inspired by vipassana meditation coming from Buddhism, on a variety of symptoms related to chronic pain, anxiety disorders, depression versus well-being and several other medical symptoms like fatigue, fibromyalgia and insomnia [306, 309–326]. Although these stress-counteracting approaches show large differences in the way mindfulness is practiced [319], and are drifting away from classical mindfulness [327], obvious effects on the neuronal substrate have been observed, using a variety of techniques [314, 319, 328–336]. We conclude that meditation is an effective means to manage stress [280] and that β-END is extensively involved in the balance between the positive and relaxing effects of activation of the reward circuitry and the negative consequences of physical problems, pain and (chronic) stress [337], shifting the balance in a positive direction, in accordance with our hypothesis as developed by Panksepp [59, 338–344]. The euphoric state, with a total neglect of bodily and environmental cues including complete suppression of pain [71, 79] can be considered as the most extreme condition in the regular balance between the brain-states and circuitries related to stress and stress-relief.

### Pain control mechanisms

The extremely potent analgesic effects of β-END were discovered early, [350–355]and during the late seventies, eighties and early nineties an extensive series of publications appeared in which CSF-levels of β-END were measured and correlated with the pain levels experienced by patients and experimental animals under a variety of painful conditions. This surge of interest emerged from the finding that the analgesic effects of β-END were obvious only after icv-, and not after iv-administration [348, 351–355] This difference illustrated clearly the relative effects of the rather impenetrable blood–brain-barrier combined with the almost 3 times shorter half-life of β-END in the blood versus in the CSF (about 37 min vs. about 97 min; [355]. Despite the originally high expectations, it turned out that the correlations of central β-END levels with chronic pain states were weak or negligible [356–364] with a few exceptions related to abdominal pain and migraine, where CSF-levels tended to be lower than normal [365–372]. While these essentially negative findings make it impossible to use CSF-levels of β-END as a parameter signaling pain perception, there is no reason to conclude that CSF-β-END levels do not play a role in pain control [373].

From the beginning it was established that not only icv-administration of β-END [354, 355] but also electro- or magnetic stimulation [346, 374–397], electro-acupuncture [380–397] as well as physical exercise or therapy [398, 399] induced strong analgesic effects, by elevating the levels of β-END in the CSF. Since acute painful stimuli also raise these CSF levels considerably [170, 359, 375, 400, 401], it is fully clear that the central release of β-END forms part of an antinociceptive system controlling pain [71, 79, 402, 403]. It has been observed that mindfulness meditation modulates pain perception as well, e.g. [320]. This antinociceptive system includes the arcuate hypothalamic nucleus (ARH), with its content of POMC-neurons, the periaqueductal gray region [390, 404, 405] and several caudal brainstem areas, including the caudal raphe nuclei, from where 5-HT projections descend into the spinal cord [402, 403] Many experimental data show, however, that this system is not limited to a set of (partially reciprocal) neuronal connections but that β-END, released into the CSF to go with the flow [14, 404, 405], plays an important modulating role.

As early as 1982, James Henry argued that the variety of conditions under which β-END is released and the fact that these conditions exert effects on a number of systems together, requires a global activation of opiate receptors throughout the CNS [71]. At the time, blood-borne opioid hormone, released by the pituitary or a pituitary-controlled peripheral gland to enter the CNS and the CSF, appeared to be the most appropriate candidate to induce such a generalized response [71, 79]. Later studies, however, showed that β-END can also be released locally in peripheral tissues, to control (local) pain [177, 179, 181, 406–408]. Peripheral levels of β-END may influence the spinal cord [5, 71], but brain-CSF levels arise from the arcuate hypothalamic nucleus (ARH) as well as from numerous terminals surrounding the ventricular spaces and are sufficiently high to induce the central effects. The following findings provide additional evidence.

Extracellular levels of β-END in the ARH show two- to fourfold increases upon painful or 5-HT stimulation. While this release may potentially activate all neighboring POMC neurons, the destination will be the adjoining CSF [404, 405], to ‘go with the flow’. The flowing CSF may collect additional POMC and β-END from the many varicose fibers running subependymally alongside the ventricular system. It is therefore not surprising that high levels of β-END have been measured in the CSF after stimulating its release by a painful or an electrical stimulus [170, 374, 375, 377, 409–412] CSF-levels increased 20-fold, after this central release and the analgesic effects were clearly correlated with the duration and the increase of the levels. In 2001 Shen [379] reported an elucidating rabbit experiment. CSF from one animal, after 30 min of electro-acupuncture, was infused into the lateral ventricle of a naïve recipient rabbit. The analgesic effect was observed in the recipient rabbit, showing that volume transmission via the CSF can indeed be effective. More recently, Zubrzycka and Janecka, showed in an experiment involving both tooth pulp- and central gray electrical stimulation, that β-END was released into the CSF after tooth pulp stimulation and this release could be inhibited by the PAG-stimulation [405, 412]. They concluded that ‘endogenous β-END, released as a result of electrical tooth pulp stimulation in orofacial pain, diffuses through the cerebroventricular ependyma into the CSF and exerts a modulatory effect, mediated by μ-receptors, altering the properties of neurons in the trigeminal sensory nuclei, interneurons, and motoneurons of the hypoglossal nerve’ [405].

Additional evidence comes from intranasal (IN-) application studies, showing that a large variety of substances follow direct nose-to-brain pathways to enter the brain cavity and the CSF compartment, generally within a few minutes [22]. For β-END itself the effects of IN-administration have been hardly reported, probably because of its adverse effects on the nasal epithelium [413, 414] Only in an older monkey study clear-cut effects of IN-β-END were reported, in this case on induced prolactin levels [415]. Several substances supposed to elevate central β-END levels: desmopressin, [416] and calcitonin [417, 418] have been tried with variable results and without measurement of CSF-levels. Intranasally applied morphine, however, has been shown to reach the ventricular system of rodents within minutes with a clear distribution advantage over the intravenous and especially oral administration levels [419, 420]. Peripheral levels apparently played no role in the elevated brain levels. Several substances are currently applied intranasally for clinical pain relief, especially fentanyl [416, 421–426] and the opioid system and the CSF as the transport medium seem to be always involved in the effects of such substances. In addition to mechanisms introducing substances into the CSF, it has also been observed that specific ependymal cells and other neurons are able to take up and transport specific substances from the CSF, towards the soma of neurons, frequently located far away from the ventricular surfaces, where they may elicit responses leading to changes in gene expression [427–429].

Already mentioned were some studies in which transport via the CSF seems to be the only possible explanation of the observed effects. Yadid *et al*. observed a strong reduction of pain behaviors after transplantation of adrenal medullary cells into the subarachnoid space of the spinal cord [430, 431] but there were several reasons to assume that the observed effects were not the local effects of the transplant. In 2000, Yadid *et al*. showed the involvement of central β-END mechanisms and the arcuate nucleus in the observed analgesia, apparently by releasing β-END via the CSF as a transporter of messages [411]. In another interesting study, Finegold *et al*. transferred genes to the meninges surrounding the spinal cord, upon which pia mater cells started to produce β-END [432]. This pia mater-release of β-END had a clear analgesic effect on an inflammation model of persistent pain. Their paracrine paradigm for the treatment of chronic pain shows that substances released in the CSF become functional when transported via the CSF. In conclusion, β-END plays a complicated but major role in the mechanisms controlling pain, and transport via the CSF (volume transmission) forms an essential part in these effects [14]. This role is in full agreement with the rewarding and anti-stress roles put forward in the preceding sections of this review.

## Other effects of β-END

In order to limit the scope of the present review, additional behavioral, functional and clinical effects of β-END will not be discussed in detail. Such effects have been reported, for example, on the mechanisms of intestinal transit [439] cardiovascular control [103, 122, 434] and the growth and metastasis of mammary tumor cells [435], but also on the immune system, complex arthritic inflammatory syndromes, fibromyalgia and cerebral infarction [180, 271, 436–448]. An extra complication is that the control and effects of β-END are extensively related to a variety of other humoral and neuromodulatory factors, like serotonin, involved in syndrome such as schizophrenia and depression [449, 450]. For that reason, Hegadoren *et al*. called attention to a possible role of β-END in the pathophysiology of major depression [337]. They reviewed the multiple interactive links between serotonin, β-END and the HPA-axis involved in major depression. Such interactions are of great importance in view of the hypothesis that β-END is involved in anti-stress mechanisms and well-being. Perhaps the involvement of β-END in depression gives this mind-state a considerable, but undesirable, degree of stability. In that case, the stability of a depression has to be considered as an unpleasant side effect. A discussion of these possibilities is beyond the scope of the present review.

One interaction effect deserves some special attention, namely with the neuropeptide oxytocin (OT), because this peptide plays a major role in positive social interactions and because of it may be released into the CSF to influence distant brain areas by going with the flow [3, 4, 22]. In 1989 Melrose and Knigge studied horse brains and proposed evolutionary relationships for POMC, oxytocin (OT) and vasopressin (AVP) neurons, all of them surrounding the ventricular system and equipped with an extensive set of mutual connections [451]. The remarkable co-existence of opiocortin and corticotropin-releasing factor immunoreactive CRF-IR projections surrounding the ventricular system, as observed from the earliest studies [452, 453], contributes to the suggestion of mutual interaction effects. The fact that each of these neuropeptides shows facilitatory or inhibitory effects on behavior as well as physiological mechanisms like feeding, sex, aggression, pain, reward, (anti)stress and social relationships like maternal and pair-bonding behavior, makes the conclusion unavoidable that these neuropeptides must operate in close mutual interactive relationships. Many behavioral and physiological studies support the existence of these functional relationships [14, 182, 454–479]. Concerning the focus of the present review, central mechanisms using the CSF for transport, readers are also referred to [3–5, 14, 22, 480, 481].

## Conclusions

Summarizing the effects of β-END on brain and behavior as described in the literature, they seem to be separable into 2 categories. On the one hand, β-END, released into the CSF, to go with the flow, may have far-reaching effects on distant brain regions involved in a variety of behaviors, related to reward mechanisms and motivational and mental states. This is the global effect with a tendency towards stress-reduction, leading to a sense of well-being by homeostatic balance and behavioral stability. On the other hand, and in addition to this state-transition- effect, local administration of β-END in specific brain areas like amygdala or hypothalamus induces specific inhibitory effects on transitions of the behavioral sequence thereby preventing the occurrence of a specific goal. A funnel-model has been introduced to describe the successive phases of a behavioral sequence. The question as to how far the specific behavioral effects always support the global effect or have to be considered as specific local mechanisms controlling specific behavioral sequences needs further attention and research. Additional experiments with local manipulation of β-END levels in specific brain regions are needed to shed more light on the complex global and specific effects of β-END on the CNS, on behavior and behavioral transitions.

## Abbreviations

α-MSH: Alpha-melanocyte-stimulating hormone
ARH: Arcuate hypothalamic nucleus
AVP: Vasopressin
β-END: Beta-endorphin
CRH: Corticotropin-releasing hormone
CNS: Central nervous system
CSF: Cerebrospinal fluid
EB: Estradiol benzoate
GABA: Gamma-aminobutyric acid
GAL: Galanin
GnRH: Gonadotropin-releasing hormone
HPA-: Hypothalamus-pituitary-adrenal-
5-HT: Serotonin
icv: Intracerebroventricular
IN-: Intranasal-
-IR: -immunoreactive
KO-: Knock-out-
LH: Luteinizing hormone
MC3/4: Melanocortin receptor types
MeA: Medial amygdala
MPOA: Medial preoptic area
NPY: Neuropeptide Y
OT: Oxytocin
PAG: Periaqueductal gray
POMC: Proopiomelanocortin
THC: Tetrahydrocannabinol
VMH: Ventromedial hypothalamic nucleus
VT: Volume transmission.

## References

[1] Turing A (1937). On computable numbers, with an application to theEntscheidungsproblem. Proc London Mathematical Soc, Series 2.

[2] Salzman CD, Fusi S (2010). Emotion, cognition, and mental state representation in amygdala and prefrontal cortex. Annu Rev Neurosci.

[3] Veening JG, Barendregt HP (2010). The regulation of brain states by neuroactive substances distributed via the cerebrospinal fluid; a review. Cerebrospinal Fluid Res.

[4] Veening JG, de Jong T, Barendregt HP (2010). Oxytocin-messages via the cerebrospinal fluid: behavioral effects; a review. Physiol Behav.

[5] Veening JG, Gerrits PO, Barendregt HP (2012). Volume transmission of beta-endorphin via the cerebrospinal fluid; a review. Fluids Barriers CNS.

[6] Agnati LF, Bjelke B, Fuxe K (1995). Volume versus wiring transmission in the brain: a new theoretical frame for neuropsychopharmacology. Med Res Rev.

[7] Agnati LF, Cortelli P, Biagini G, Bjelke B, Fuxe K (1994). Different classes of volume transmission signals exist in the central nervous system and are affected by metabolic signals, temperature gradients and pressure waves. Neuroreport.

[8] Agnati LF, Fuxe K (2000). Volume transmission as a key feature of information handling in the central nervous system possible new interpretative value of the Turing’s B-type machine. Prog Brain Res.

[9] Agnati LF, Fuxe K, Zoli M, Ozini I, Toffano G, Ferraguti F (1986). A correlation analysis of the regional distribution of central enkephalin and beta-endorphin immunoreactive terminals and of opiate receptors in adult and old male rats. Evidence for the existence of two main types of communication in the central nervous system: the volume transmission and the wiring transmission. Acta Physiol Scand.

[10] Fuxe K, Borroto-Escuela DO, Romero-Fernandez W, Ciruela F, Manger P, Leo G (2012). On the role of volume transmission and receptor-receptor interactions in social behaviour: focus on central catecholamine and oxytocin neurons. Brain Res.

[11] Fuxe K, Rivera A, Jacobsen KX, Hoistad M, Leo G, Horvath TL (2005). Dynamics of volume transmission in the brain. Focus on catecholamine and opioid peptide communication and the role of uncoupling protein 2. J Neural Transm.

[12] Agnati LF, Guidolin D, Guescini M, Genedani S, Fuxe K (2010). Understanding wiring and volume transmission. Brain Res Rev.

[13] Agnati LF, Leo G, Zanardi A, Genedani S, Rivera A, Fuxe K (2006). Volume transmission and wiring transmission from cellular to molecular networks: history and perspectives. Acta Physiol (Oxf).

[14] Fuxe K, Borroto-Escuela DO, Romero-Fernandez W, Zhang WB, Agnati LF (2013). Volume transmission and its different forms in the central nervous system. Chin J Integr Med.

[15] Fuxe K, Dahlstrom AB, Jonsson G, Marcellino D, Guescini M, Dam M (2010). The discovery of central monoamine neurons gave volume transmission to the wired brain. Prog Neurobiol.

[16] Agnati LF, Zoli M, Stromberg I, Fuxe K (1995). Intercellular communication in the brain: wiring versus volume transmission. Neuroscience.

[17] Malpaux B, Daveau A, Maurice-Mandon F, Duarte G, Chemineau P (1998). Evidence that melatonin acts in the premammillary hypothalamic area to control reproduction in the ewe: presence of binding sites and stimulation of luteinizing hormone secretion by in situ microimplant delivery. Endocrinology.

[18] Skinner DC, Malpaux B (1999). High melatonin concentrations in third ventricular cerebrospinal fluid are not due to Galen vein blood recirculating through the choroid plexus. Endocrinology.

[19] Sliwowska JH, Billings HJ, Goodman RL, Coolen LM, Lehman MN (2004). The premammillary hypothalamic area of the ewe: anatomical characterization of a melatonin target area mediating seasonal reproduction. Biol Reprod.

[20] Tricoire H, Locatelli A, Chemineau P, Malpaux B (2002). Melatonin enters the cerebrospinal fluid through the pineal recess. Endocrinology.

[21] Tricoire H, Moller M, Chemineau P, Malpaux B (2003). Origin of cerebrospinal fluid melatonin and possible function in the integration of photoperiod. Reprod Suppl.

[22] Veening JG, Olivier B (2013). Intranasal administration of oxytocin: behavioral and clinical effects, a review. Neurosci Biobehav Rev.

[23] Barendregt H, Raffone A, Cooper B, van Leeuwen J (2013). Conscious cognition as a discrete, deterministic, and universal Turing Machine process. The selected works of AM Turing.

[24] Teuscher C (2002). Turing’s Connectionism. An investigation of Neural Network Architectures.

[25] Turing AM, Meltzer B, Michie D (1969). Intelligent machinery. Machine intelligence. Volume 5.

[26] Zylberberg A, Dehaene S, Roelfsema PR, Sigman M (2011). The human Turing machine: a neural framework for mental programs. Trends in Cognnitive Sci.

[27] VanRullen R, Koch C (2003). Is perception discrete or continuous?. Trends in Cognnitive Sci.

[28] Pascual-Marqui RD, Michel RD, Lehmann D (1995). Segmentation of brain electrical activity into microstates. IEEE Trans Biomed Eng.

[29] Veening JG (1975). Behavioural functions of the VMH in the rat: an ethological approach.

[30] Veening JG (1992). Brain and behaviour: morphological and functional aspects of the hypothalamus in the rat. Eur J Morphol.

[31] Veening JG, Coolen LM (2014). Neural mechanisms of sexual behavior in the male rat: Emphasis on ejaculation-related circuits. Pharmacol Biochem Behav.

[32] Coolen LM (2005). Neural control of ejaculation. J Comp Neurol.

[33] Coolen LM, Allard J, Truitt WA, McKenna KE (2004). Central regulation of ejaculation. Physiol Behav.

[34] Coolen LM, Hull EM (2004). Male sexual function. Physiol Behav.

[35] Ahmed B, Kastin AJ, Banks WA, Zadina JE (1994). CNS effects of peptides: a cross-listing of peptides and their central actions published in the journal Peptides, 1986–1993. Peptides.

[36] Bodnar RJ (2012). Endogenous opiates and behavior: 2011. Peptides.

[37] Font L, Lujan MA, Pastor R (2013). Involvement of the endogenous opioid system in the psychopharmacological actions of ethanol: the role of acetaldehyde. Front Behav Neurosci.

[38] Herbert J (1993). Peptides in the limbic system: neurochemical codes for co-ordinated adaptive responses to behavioural and physiological demand. Prog Neurobiol.

[39] Olson GA, Olson RD, Kastin AJ (1983). Endogenous opiates: 1982. Peptides.

[40] Olson GA, Olson RD, Kastin AJ (1984). Endogenous opiates: 1983. Peptides.

[41] Olson GA, Olson RD, Kastin AJ (1985). Endogenous opiates: 1984. Peptides.

[42] Olson GA, Olson RD, Kastin AJ (1986). Endogenous opiates: 1985. Peptides.

[43] Olson GA, Olson RD, Kastin AJ (1987). Endogenous opiates: 1986. Peptides.

[44] Olson GA, Olson RD, Kastin AJ (1989). Endogenous opiates: 1988. Peptides.

[45] Olson GA, Olson RD, Kastin AJ (1989). Endogenous opiates: 1987. Peptides.

[46] Olson GA, Olson RD, Kastin AJ (1990). Endogenous opiates: 1989. Peptides.

[47] Olson GA, Olson RD, Kastin AJ (1991). Endogenous opiates: 1990. Peptides.

[48] Olson GA, Olson RD, Kastin AJ (1992). Endogenous opiates: 1991. Peptides.

[49] Olson GA, Olson RD, Kastin AJ (1993). Endogenous opiates: 1992. Peptides.

[50] Olson GA, Olson RD, Kastin AJ (1994). Endogenous opiates: 1993. Peptides.

[51] Olson GA, Olson RD, Kastin AJ (1995). Endogenous opiates: 1994. Peptides.

[52] Olson GA, Olson RD, Kastin AJ (1996). Endogenous opiates: 1995. Peptides.

[53] Olson GA, Olson RD, Kastin AJ (1997). Endogenous opiates: 1996. Peptides.

[54] Olson GA, Olson RD, Kastin AJ, Coy DH (1979). Endogenous opiates: through 1978. Neurosci Biobehav Rev.

[55] Olson GA, Olson RD, Kastin AJ, Coy DH (1980). Endogenous opiates: 1979(1). Peptides.

[56] Olson GA, Olson RD, Kastin AJ, Coy DH (1981). Endogenous opiates: 1980. Peptides.

[57] Olson GA, Olson RD, Kastin AJ, Coy DH (1982). Endogenous opiates: 1981. Peptides.

[58] Olson GA, Olson RD, Vaccarino AL, Kastin AJ (1998). Endogenous opiates: 1997. Peptides.

[59] Panksepp J (1986). The neurochemistry of behavior. Annu Rev Psychol.

[60] Roy A, Roy M, Deb S, Unwin G, Roy A (2014). Are opioid antagonists effective in attenuating the core symptoms of autism spectrum conditions in children: a systematic review. J Intellect Disabil Res.

[61] Sarkar DK, Zhang C (2013). Beta-endorphin neuron regulates stress response and innate immunity to prevent breast cancer growth and progression. Vitam Horm.

[62] Vaccarino AL, Olson GA, Olson RD, Kastin AJ (1999). Endogenous opiates: 1998. Peptides.

[63] Zhao K (2013). Acupuncture for the treatment of insomnia. Int Rev Neurobiol.

[64] Benedetti F: **Placebo and endogenous mechanisms of analgesia.** *Handb Exp Pharmacol* 2007, (177)**:**393–413. Review. PubMed PMID: 17087131 10.1007/978-3-540-33823-9_14 17087131

[65] Butcher BE, Carmody JJ (2012). Sex differences in analgesic response to ibuprofen are influenced by expectancy: a randomized, crossover, balanced placebo-designed study. Eur J Pain.

[66] Jaksic N, Aukst-Margetic B, Jakovljevic M (2013). Does personality play a relevant role in the placebo effect?. Psychiatr Danub.

[67] Lemoine P (2011). The placebo mystery or neurobiology of the soul. Bull Acad Natl Med.

[68] Mommaerts JL, Devroey D (2012). The placebo effect: how the subconscious fits in. Perspect Biol Med.

[69] Zubieta JK, Stohler CS (2009). Neurobiological mechanisms of placebo responses. Ann N Y Acad Sci.

[70] Diederich NJ, Goetz CG (2008). The placebo treatments in neurosciences: New insights from clinical and neuroimaging studies. Neurology.

[71] Henry JL (1982). Circulating opioids: possible physiological roles in central nervous function. Neurosci Biobehav Rev.

[72] Akil H, Watson SJ, Young E, Lewis ME, Khachaturian H, Walker JM (1984). Endogenous opioids: biology and function. Annu Rev Neurosci.

[73] Izquierdo I, Netto CA (1985). The brain beta-endorphin system and behavior: the modulation of consecutively and simultaneously processed memories. Behav Neural Biol.

[74] Izquierdo I, Netto CA (1985). Role of beta-endorphin in behavioral regulation. Ann N Y Acad Sci.

[75] Izquierdo I, Netto CA, Carrasco MA, Dias RD, Volkmer N (1985). The course of the decrease of hypothalamic beta-endorphin induced by training, and the development of the effect of beta-endorphin on the retrieval of inhibitory avoidance in rats. Braz J Med Biol Res.

[76] Keverne EB, Martensz ND, Tuite B (1989). Beta-endorphin concentrations in cerebrospinal fluid of monkeys are influenced by grooming relationships. Psychoneuroendocrinology.

[77] Martensz ND, Vellucci SV, Keverne EB, Herbert J (1986). beta-Endorphin levels in the cerebrospinal fluid of male talapoin monkeys in social groups related to dominance status and the luteinizing hormone response to naloxone. Neuroscience.

[78] Roberts AC, Martensz ND, Hastings MH, Herbert J (1987). The effects of castration, testosterone replacement and photoperiod upon hypothalamic beta-endorphin levels in the male Syrian hamster. Neuroscience.

[79] Henry JL (1986). Role of circulating opioids in the modulation of pain. Ann N Y Acad Sci.

[80] Hughes AM, Everitt BJ, Herbert J (1987). Selective effects of beta-endorphin infused into the hypothalamus, preoptic area and bed nucleus of the stria terminalis on the sexual and ingestive behaviour of male rats. Neuroscience.

[81] Hughes AM, Everitt BJ, Herbert J (1988). The effects of simultaneous or separate infusions of some pro-opiomelanocortin-derived peptides (beta-endorphin, melanocyte stimulating hormone, and corticotrophin-like intermediate polypeptide) and their acetylated derivatives upon sexual and ingestive behaviour of male rats. Neuroscience.

[82] Hughes AM, Everitt BJ, Herbert J (1990). Comparative effects of preoptic area infusions of opioid peptides, lesions and castration on sexual behaviour in male rats: studies of instrumental behaviour, conditioned place preference and partner preference. Psychopharmacology (Berl).

[83] McGregor A, Herbert J (1992). The effects of beta-endorphin infusions into the amygdala on visual and olfactory sensory processing during sexual behaviour in the male rat. Neuroscience.

[84] McGregor A, Herbert J (1992). Specific effects of beta-endorphin infused into the amygdala on sexual behaviour in the male rat. Neuroscience.

[85] Stavy M, Herbert J (1989). Differential effects of beta-endorphin infused into the hypothalamic preoptic area at various phases of the male rat’s sexual behaviour. Neuroscience.

[86] Mercer AJ, Stuart RC, Attard CA, Otero-Corchon V, Nillni EA, Low MJ (2014). Temporal changes in nutritional state affect hypothalamic POMC peptide levels independently of leptin in adult male mice. Am J Physiol Endocrinol Metab.

[87] Cheung CC, Clifton DK, Steiner RA (1997). Proopiomelanocortin neurons are direct targets for leptin in the hypothalamus. Endocrinology.

[88] Pritchard LE, White A (2007). Neuropeptide processing and its impact on melanocortin pathways. Endocrinology.

[89] Pritchard LE, Oliver RL, McLoughlin JD, Birtles S, Lawrence CB, Turnbull AV (2003). Proopiomelanocortin-derived peptides in rat cerebrospinal fluid and hypothalamic extracts: evidence that secretion is regulated with respect to energy balance. Endocrinology.

[90] Millington GW (2007). The role of proopiomelanocortin (POMC) neurones in feeding behaviour. Nutr Metab (Lond).

[91] Ma J, Xia J, Miele L, Sarkar FH, Wang Z: **Notch signaling pathway in pancreatic cancer progression.** *Pancreat Disord Ther* 2013.,**30**(114)**:** pii: 1000114. PubMed PMID: 24027656; PubMed Central PMCID: PMC3767173 PMC3767173 24027656

[92] Lee M, Kim A, Conwell IM, Hruby V, Mayorov A, Cai M (2008). Effects of selective modulation of the central melanocortin-3-receptor on food intake and hypothalamic POMC expression. Peptides.

[93] Kim MS, Rossi M, Abusnana S, Sunter D, Morgan DG, Small CJ (2000). Hypothalamic localization of the feeding effect of agouti-related peptide and alpha-melanocyte-stimulating hormone. Diabetes.

[94] Zheng H, Patterson LM, Rhodes CJ, Louis GW, Skibicka KP, Grill HJ (2010). A potential role for hypothalamomedullary POMC projections in leptin-induced suppression of food intake. Am J Physiol Regul Integr Comp Physiol.

[95] Seeley RJ, Drazen DL, Clegg DJ (2004). The critical role of the melanocortin system in the control of energy balance. Annu Rev Nutr.

[96] de Backer MW, la Fleur SE, Brans MA, van Rozen AJ, Luijendijk MC, Merkestein M (2011). Melanocortin receptor-mediated effects on obesity are distributed over specific hypothalamic regions. Int J Obes (Lond).

[97] Werner L, Muller-Fielitz H, Ritzal M, Werner T, Rossner M, Schwaninger M (2012). Involvement of doublecortin-expressing cells in the arcuate nucleus in body weight regulation. Endocrinology.

[98] Kim JW, Park TJ, Ryu DD, Kim JY (2000). High cell density culture of Yarrowia lipolytica using a one-step feeding process. Biotechnol Prog.

[99] Petervari E, Balasko M, Garami A, Soos S, Szekely M (2009). Suppression of food intake by intracerebroventricular injection of alpha-MSH varies with age in rats. Acta Physiol Hung.

[100] Zheng H, Patterson LM, Phifer CB, Berthoud HR (2005). Brain stem melanocortinergic modulation of meal size and identification of hypothalamic POMC projections. Am J Physiol Regul Integr Comp Physiol.

[101] Dutta J, Tripathi S, Dutta PK (2012). Progress in antimicrobial activities of chitin, chitosan and its oligosaccharides: a systematic study needs for food applications. Food Sci Technol Int.

[102] Barfield ET, Moser VA, Hand A, Grisel JE (2013). beta-endorphin modulates the effect of stress on novelty-suppressed feeding. Front Behav Neurosci.

[103] Skibicka KP, Grill HJ (2009). Hypothalamic and hindbrain melanocortin receptors contribute to the feeding, thermogenic, and cardiovascular action of melanocortins. Endocrinology.

[104] Dutia R, Meece K, Dighe S, Kim AJ, Wardlaw SL (2012). beta-Endorphin antagonizes the effects of alpha-MSH on food intake and body weight. Endocrinology.

[105] Sanger DJ, McCarthy PS (1981). Increased food and water intake produced in rats by opiate receptor agonists. Psychopharmacology (Berl).

[106] Sanger DJ, McCarthy PS, Metcalf G (1981). The effects of opiate antagonists on food intake are stereospecific. Neuropharmacology.

[107] Morley JE, Levine AS, Murray SS, Kneip J (1982). Peptidergic regulation of norepinephrine induced feeding. Pharmacol Biochem Behav.

[108] Morley JE, Levine AS, Plotka ED, Seal US (1983). The effect of naloxone on feeding and spontaneous locomotion in the wolf. Physiol Behav.

[109] Morley JE, Bartness TJ, Gosnell BA, Levine AS (1985). Peptidergic regulation of feeding. Int Rev Neurobiol.

[110] Grandison L, Guidotti A (1977). Stimulation of food intake by muscimol and beta endorphin. Neuropharmacology.

[111] Sanger DJ (1981). Endorphinergic mechanisms in the control of food and water intake. Appetite.

[112] Morley JE, Levine AS, Grace M, Kniep J (1982). An investigation of the role of kappa opiate receptor agonists in the initiation of feeding. Life Sci.

[113] Dube MG, Horvath TL, Leranth C, Kalra PS, Kalra SP (1994). Naloxone reduces the feeding evoked by intracerebroventricular galanin injection. Physiol Behav.

[114] Wang QP, Guan JL, Nakai Y (1998). Beta-endorphinergic innervation of mu and delta receptor containing neurons in the dorsal raphe nucleus. Neuroreport.

[115] Wang QP, Ochiai H, Guan JL, Nakai Y (1998). Ultrastructural localization of mu-1 opioid receptor in the dorsal raphe nucleus of the rat. Synapse.

[116] Robert JJ, Orosco M, Rouch C, Cohen Y, Jacquot C (1991). Effects of dexfenfluramine and opioid peptides, alone or in combination, on food intake and brain serotonin turnover in rats. Pharmacol Biochem Behav.

[117] Yamamoto T, Sako N, Maeda S (2000). Effects of taste stimulation on beta-endorphin levels in rat cerebrospinal fluid and plasma. Physiol Behav.

[118] Matsumura S, Eguchi A, Okafuji Y, Tatsu S, Mizushige T, Tsuzuki S (2012). Dietary fat ingestion activates beta-endorphin neurons in the hypothalamus. FEBS Lett.

[119] Mizushige T, Saitoh K, Manabe Y, Nishizuka T, Taka Y, Eguchi A (2009). Preference for dietary fat induced by release of beta-endorphin in rats. Life Sci.

[120] Low MJ, Hayward MD, Appleyard SM, Rubinstein M (2003). State-dependent modulation of feeding behavior by proopiomelanocortin-derived beta-endorphin. Ann N Y Acad Sci.

[121] Appleyard SM, Hayward M, Young JI, Butler AA, Cone RD, Rubinstein M (2003). A role for the endogenous opioid beta-endorphin in energy homeostasis. Endocrinology.

[122] Hill C, Lapanowski K, Dunbar JC (2002). The effects of beta-endorphin (beta-END) on cardiovascular and behavioral dynamics in conscious rats. Brain Res Bull.

[123] Carr KD, Simon EJ (1983). The role of opioids in feeding and reward elicited by lateral hypothalamic electrical stimulation. Life Sci.

[124] Carr KD, Bak TH (1990). Rostral and caudal ventricular infusion of antibodies to dynorphin A(1–17) and dynorphin A(1–8): effects on electrically-elicited feeding in the rat. Brain Res.

[125] Carr KD, Wolinsky TD (1994). Regulation of feeding by multiple opioid receptors in cingulate cortex; follow-up to an in vivo autoradiographic study. Neuropeptides.

[126] Papadouka V, Carr KD (1994). The role of multiple opioid receptors in the maintenance of stimulation-induced feeding. Brain Res.

[127] de Ruiter L, Wiepkema PR, Veening JG (1974). Models of behavior and the hypothalamus. Prog Brain Res.

[128] Wiepkema PR (1971). Behavioural factors in the regulation of food intake. Proc Nutr Soc.

[129] Wiepkema PR (1971). Positive feedbacks at work during feeding. Behaviour.

[130] Argiolas A, Melis MR (2013). Neuropeptides and central control of sexual behaviour from the past to the present: a review. Prog Neurobiol.

[131] Mahler SV, Berridge KC (2012). What and when to “want”? Amygdala-based focusing of incentive salience upon sugar and sex. Psychopharmacology (Berl).

[132] Pfaus JG, Gorzalka BB (1987). Opioids and sexual behavior. Neurosci Biobehav Rev.

[133] Pfaus JG, Kippin TE, Coria-Avila GA, Gelez H, Afonso VM, Ismail N (2012). Who, what, where, when (and maybe even why)? How the experience of sexual reward connects sexual desire, preference, and performance. Arch Sex Behav.

[134] McIntosh TK, Vallano ML, Barfield RJ (1980). Effects of morphine, beta-endorphin and naloxone on catecholamine levels and sexual behavior in the male rat. Pharmacol Biochem Behav.

[135] Dornan WA, Malsbury CW (1989). Neuropeptides and male sexual behavior. Neurosci Biobehav Rev.

[136] Meyerson BJ, Terenius L (1977). Beta-endorphin and male sexual behavior. Eur J Pharmacol.

[137] Teodorov E, Camarini R, Bernardi MM, Felicio LF (2014). Treatment with steroid hormones and morphine alters general activity, sexual behavior, and opioid gene expression in female rats. Life Sci.

[138] Parada M, Vargas EB, Kyres M, Burnside K, Pfaus JG (2012). The role of ovarian hormones in sexual reward states of the female rat. Horm Behav.

[139] Gessa GL, Paglietti E, Quarantotti BP (1979). Induction of copulatory behavior in sexually inactive rats by naloxine. Science.

[140] Myers BM, Baum MJ (1979). Facilitation by opiate antagonists of sexual performance in the male rat. Pharmacol Biochem Behav.

[141] Glick BB, Baughman WL, Jensen JN, Phoenix CH (1982). Endogenous opiate systems and primate reproduction: inability of naloxone to induce sexual activity in rhesus males. Arch Sex Behav.

[142] Fabbri A, Jannini EA, Gnessi L, Moretti C, Ulisse S, Franzese A (1989). Endorphins in male impotence: evidence for naltrexone stimulation of erectile activity in patient therapy. Psychoneuroendocrinology.

[143] Bishop JD, Malven PV, Singleton WL, Weesner GD (1999). Hormonal and behavioral correlates of emotional states in sexually trained boars. J Anim Sci.

[144] Carani C, Bancroft J, Del Rio G, Granata AR, Facchinetti F, Marrama P (1990). The endocrine effects of visual erotic stimuli in normal men. Psychoneuroendocrinology.

[145] Daly RC, Su TP, Schmidt PJ, Pickar D, Murphy DL, Rubinow DR (2001). Cerebrospinal fluid and behavioral changes after methyltestosterone administration: preliminary findings. Arch Gen Psychiatry.

[146] Szechtman H, Hershkowitz M, Simantov R (1981). Sexual behavior decreases pain sensitivity and stimulated endogenous opioids in male rats. Eur J Pharmacol.

[147] Forsberg G, Wiesenfeld-Hallin Z, Eneroth P, Sodersten P (1987). Sexual behavior induces naloxone-reversible hypoalgesia in male rats. Neurosci Lett.

[148] Meyerson BJ (1981). Comparison of the effects of beta-endorphin and morphine on exploratory and socio-sexual behaviour in the male rat. Eur J Pharmacol.

[149] Burkett JP, Spiegel LL, Inoue K, Murphy AZ, Young LJ (2011). Activation of mu-opioid receptors in the dorsal striatum is necessary for adult social attachment in monogamous prairie voles. Neuropsychopharmacology.

[150] McGrady AV (1984). Effects of psychological stress on male reproduction: a review. Arch Androl.

[151] Taylor GT, Weiss J, Rupich R (1987). Male rat behavior, endocrinology and reproductive physiology in a mixed-sex, socially stressful colony. Physiol Behav.

[152] Retana-Marquez S, Salazar ED, Velazquez-Moctezuma J (1996). Effect of acute and chronic stress on masculine sexual behavior in the rat. Psychoneuroendocrinology.

[153] Sato Y, Suzuki N, Horita H, Wada H, Shibuya A, Adachi H (1996). Effects of long-term psychological stress on sexual behavior and brain catecholamine levels. J Androl.

[154] Brotto LA, Gorzalka BB, LaMarre AK (2001). Melatonin protects against the effects of chronic stress on sexual behaviour in male rats. Neuroreport.

[155] Cameron N, Erskine MS (2003). c-FOS expression in the forebrain after mating in the female rat is altered by adrenalectomy. Neuroendocrinology.

[156] Gonzalez-Mariscal G, Gomora P, Beyer C (1994). Participation of opiatergic, GABAergic, and serotonergic systems in the expression of copulatory analgesia in male rats. Pharmacol Biochem Behav.

[157] D’Amato FR, Pavone F (2012). Modulation of nociception by social factors in rodents: contribution of the opioid system. Psychopharmacology (Berl).

[158] Burgdorf J, Panksepp J (2001). Tickling induces reward in adolescent rats. Physiol Behav.

[159] Van Ree JM, Niesink RJ, Van Wolfswinkel L, Ramsey NF, Kornet MM, Van Furth WR (2000). Endogenous opioids and reward. Eur J Pharmacol.

[160] van Furth WR, van Emst MG, van Ree JM (1995). Opioids and sexual behavior of male rats: involvement of the medial preoptic area. Behav Neurosci.

[161] Van Furth WR, Van Ree JM (1996). Appetitive sexual behavior in male rats: 2. sexual reward and level-changing behavior. Physiol Behav.

[162] van Furth WR, van Ree JM (1996). Sexual motivation: involvement of endogenous opioids in the ventral tegmental area. Brain Res.

[163] Agmo A, Gomez M (1993). Sexual reinforcement is blocked by infusion of naloxone into the medial preoptic area. Behav Neurosci.

[164] Mitchell JB, Gratton A (1991). Opioid modulation and sensitization of dopamine release elicited by sexually relevant stimuli: a high speed chronoamperometric study in freely behaving rats. Brain Res.

[165] Agmo A, Berenfeld R (1990). Reinforcing properties of ejaculation in the male rat: role of opioids and dopamine. Behav Neurosci.

[166] Balfour ME, Yu L, Coolen LM (2004). Sexual behavior and sex-associated environmental cues activate the mesolimbic system in male rats. Neuropsychopharmacology.

[167] Davis BA, Fitzgerald ME, Brown JL, Amstalden KA, Coolen LM (2007). Activation of POMC neurons during general arousal but not sexual behavior in male rats. Behav Neurosci.

[168] Sinchak K, Dewing P, Ponce L, Gomez L, Christensen A, Berger M (2013). Modulation of the arcuate nucleus-medial preoptic nucleus lordosis regulating circuit: a role for GABAB receptors. Horm Behav.

[169] Agnati LF, Genedani S, Leo G, Forni A, Woods AS, Filaferro M (2007). Abeta peptides as one of the crucial volume transmission signals in the trophic units and their interactions with homocysteine. Physiological implications and relevance for Alzheimer’s disease. J Neural Transm.

[170] Bach FW, Yaksh TL (1995). Release into ventriculo-cisternal perfusate of beta-endorphin- and Met-enkephalin-immunoreactivity: effects of electrical stimulation in the arcuate nucleus and periaqueductal gray of the rat. Brain Res.

[171] van Furth WR, van Ree JM (1994). Endogenous opioids and sexual motivation and performance during the light phase of the diurnal cycle. Brain Res.

[172] van Furth WR, Wolterink G, van Ree JM (1995). Regulation of masculine sexual behavior: involvement of brain opioids and dopamine. Brain Res Brain Res Rev.

[173] Coolen LM, Fitzgerald ME, Yu L, Lehman MN (2004). Activation of mu opioid receptors in the medial preoptic area following copulation in male rats. Neuroscience.

[174] Meyerson BJ, Hoglund AU (1981). Exploratory and socio-sexual behaviour in the male laboratory rat: a methodological approach for the investigation of drug action. Acta Pharmacol Toxicol (Copenh).

[175] Wu FM, Noble RG (1986). Opiate antagonists and copulatory behavior of male hamsters. Physiol Behav.

[176] Truitt WA, Coolen LM (2002). Identification of a potential ejaculation generator in the spinal cord. Science.

[177] Alves DP, da Motta PG, Lima PP, Queiroz-Junior CM, Caliari MV, Pacheco DF (2012). Inflammation mobilizes local resources to control hyperalgesia: the role of endogenous opioid peptides. Pharmacology.

[178] Imaizumi T, Numata A, Yano C, Yoshida H, Meng P, Hayakari R (2014). ISG54 and ISG56 are induced by TLR3 signaling in U373MG human astrocytoma cells: Possible involvement in CXCL10 expression. Neurosci Res.

[179] Ishikawa T, Miyagi M, Yamashita M, Kamoda H, Eguchi Y, Arai G (2013). In-vivo transfection of the proopiomelanocortin gene, precursor of endogenous endorphin, by use of radial shock waves alleviates neuropathic pain. J Orthop Sci.

[180] Nakamoto K, Nishinaka T, Sato N, Mankura M, Koyama Y, Kasuya F (2013). Hypothalamic GPR40 signaling activated by free long chain fatty acids suppresses CFA-induced inflammatory chronic pain. PLoS One.

[181] Slominski AT, Zmijewski MA, Skobowiat C, Zbytek B, Slominski RM, Steketee JD (2012). Sensing the environment: regulation of local and global homeostasis by the skin's neuroendocrine system. Adv Anat Embryol Cell Biol.

[182] Yu JS, Zeng BY, Hsieh CL (2013). Acupuncture stimulation and neuroendocrine regulation. Int Rev Neurobiol.

[183] Zhang Y, Liu B, Ma Y, Yi J, Zhang C, Zhang Y (2014). Hantaan virus infection induces CXCL10 expression through TLR3, RIG-I, and MDA-5 pathways correlated with the disease severity. Mediators Inflamm.

[184] Coolen LM, Peters HJ, Veening JG (1996). Fos immunoreactivity in the rat brain following consummatory elements of sexual behavior: a sex comparison. Brain Res.

[185] Coolen LM, Peters HJ, Veening JG (1998). Anatomical interrelationships of the medial preoptic area and other brain regions activated following male sexual behavior: a combined fos and tract-tracing study. J Comp Neurol.

[186] Pfaus JG, Gorzalka BB (1987). Selective activation of opioid receptors differentially affects lordosis behavior in female rats. Peptides.

[187] Pfaus JG, Pendleton N, Gorzalka BB (1986). Dual effect of morphiceptin on lordosis behavior: possible mediation by different opioid receptor subtypes. Pharmacol Biochem Behav.

[188] Sirinathsinghji DJ (1984). Modulation of lordosis behavior of female rats by naloxone, beta-endorphin and its antiserum in the mesencephalic central gray: possible mediation via GnRH. Neuroendocrinology.

[189] Sirinathsinghji DJ (1986). Regulation of lordosis behaviour in the female rat by corticotropin-releasing factor, beta-endorphin/corticotropin and luteinizing hormone-releasing hormone neuronal systems in the medial preoptic area. Brain Res.

[190] Sirinathsinghji DJ, Rees LH, Rivier J, Vale W (1983). Corticotropin-releasing factor is a potent inhibitor of sexual receptivity in the female rat. Nature.

[191] Wiesner JB, Moss RL (1986). Behavioral specificity of beta-endorphin suppression of sexual behavior: differential receptor antagonism. Pharmacol Biochem Behav.

[192] Wiesner JB, Moss RL (1986). Suppression of receptive and proceptive behavior in ovariectomized, estrogen-progesterone-primed rats by intraventricular beta-endorphin: studies of behavioral specificity. Neuroendocrinology.

[193] Kubo K, Oomura Y (1986). Modulation of estrogen-activated sexual receptivity by opioid peptides. Emotions: neuronal and chemical control.

[194] Sirinathsinghji DJ, Whittington PE, Audsley A, Fraser HM (1983). beta-Endorphin regulates lordosis in female rats by modulating LH-RH release. Nature.

[195] Mills RH, Sohn RK, Micevych PE (2004). Estrogen-induced mu-opioid receptor internalization in the medial preoptic nucleus is mediated via neuropeptide Y-Y1 receptor activation in the arcuate nucleus of female rats. J Neurosci.

[196] Torii M, Kubo K, Sasaki T (1999). Facilitatory and inhibitory effects of beta-endorphin on lordosis in female rats: relation to time of administration. Horm Behav.

[197] Torii M, Kubo K, Sasaki T (1997). Differential effects of beta-endorphin and Met- and Leu-enkephalin on steroid hormone-induced lordosis in ovariectomized female rats. Pharmacol Biochem Behav.

[198] Torii M, Kubo K, Sasaki T (1996). Influence of opioid peptides on the priming action of estrogen on lordosis in ovariectomized rats. Neurosci Lett.

[199] Torii M, Kubo K, Sasaki T (1995). Naloxone and initial estrogen action to induce lordosis in ovariectomized rats: the effect of a cut between the septum and preoptic area. Neurosci Lett.

[200] Chen WP, Witkin JW, Silverman AJ (1989). beta-Endorphin and gonadotropin-releasing hormone synaptic input to gonadotropin-releasing hormone neurosecretory cells in the male rat. J Comp Neurol.

[201] Drouva SV, Epelbaum J, Tapia-Arancibia L, Laplante E, Kordon C (1981). Opiate receptors modulate LHRH and SRIF release from mediobasal hypothalamic neurons. Neuroendocrinology.

[202] Adler BA, Crowley WR (1984). Modulation of luteinizing hormone release and catecholamine activity by opiates in the female rat. Neuroendocrinology.

[203] Leranth C, MacLusky NJ, Shanabrough M, Naftolin F (1988). Immunohistochemical evidence for synaptic connections between pro-opiomelanocortin-immunoreactive axons and LH-RH neurons in the preoptic area of the rat. Brain Res.

[204] Babu GN, Marco J, Bona-Gallo A, Gallo RV (1987). Steroid-independent endogenous opioid peptide suppression of pulsatile luteinizing hormone release between estrus and diestrus in the rat estrous cycle. Brain Res.

[205] Gallo RV, Babu GN, Bona-Gallo A, Devorshak-Harvey E, Leipheimer RE, Marco J (1987). Regulation of pulsatile luteinizing hormone release during the estrous cycle and pregnancy in the rat. Adv Exp Med Biol.

[206] Martensz ND (1985). Changes in the processing of beta-endorphin in the hypothalamus and pituitary gland of female rats during sexual maturation. Neuroscience.

[207] Kawano H, Masuko S (2000). Beta-endorphin-, adrenocorticotrophic hormone- and neuropeptide y-containing projection fibers from the arcuate hypothalamic nucleus make synaptic contacts on to nucleus preopticus medianus neurons projecting to the paraventricular hypothalamic nucleus in the rat. Neuroscience.

[208] Berglund LA, Simpkins JW (1988). Alterations in brain opiate receptor mechanisms on proestrous afternoon. Neuroendocrinology.

[209] Berglund LA, Derendorf H, Simpkins JW (1988). Desensitization of brain opiate receptor mechanisms by gonadal steroid treatments that stimulate luteinizing hormone secretion. Endocrinology.

[210] Leranth C, MacLusky NJ, Shanabrough M, Naftolin F (1988). Catecholaminergic innervation of luteinizing hormone-releasing hormone and glutamic acid decarboxylase immunopositive neurons in the rat medial preoptic area. An electron-microscopic double immunostaining and degeneration study. Neuroendocrinology.

[211] Hammer RP, Zhou L, Cheung S (1994). Gonadal steroid hormones and hypothalamic opioid circuitry. Horm Behav.

[212] Sinchak K, Romeo HE, Micevych PE (2006). Site-specific estrogen and progestin regulation of orphanin FQ/nociceptin and nociceptin opioid receptor mRNA expression in the female rat limbic hypothalamic system. J Comp Neurol.

[213] Sinchak K, Shahedi K, Dewing P, Micevych P (2005). Sexual receptivity is reduced in the female mu-opioid receptor knockout mouse. Neuroreport.

[214] Sinchak K, Micevych P (2003). Visualizing activation of opioid circuits by internalization of G protein-coupled receptors. Mol Neurobiol.

[215] Micevych PE, Rissman EF, Gustafsson JA, Sinchak K (2003). Estrogen receptor-alpha is required for estrogen-induced mu-opioid receptor internalization. J Neurosci Res.

[216] Sinchak K, Micevych PE (2001). Progesterone blockade of estrogen activation of mu-opioid receptors regulates reproductive behavior. J Neurosci.

[217] Sinchak K, Mills RH, Eckersell CB, Micevych PE (2004). Medial preoptic area delta-opioid receptors inhibit lordosis. Behav Brain Res.

[218] Jirikowski GF, Merchenthaler I, Rieger GE, Stumpf WE (1986). Estradiol target sites immunoreactive for beta-endorphin in the arcuate nucleus of rat and mouse hypothalamus. Neurosci Lett.

[219] Thornton JE, Loose MD, Kelly MJ, Ronnekleiv OK (1994). Effects of estrogen on the number of neurons expressing beta-endorphin in the medial basal hypothalamus of the female guinea pig. J Comp Neurol.

[220] Miller MM, Tousignant P, Yang U, Pedvis S, Billiar RB (1995). Effects of age and long-term ovariectomy on the estrogen-receptor containing subpopulations of beta-endorphin-immunoreactive neurons in the arcuate nucleus of female C57BL/6J mice. Neuroendocrinology.

[221] Cheung S, Hammer RP (1995). Gonadal steroid hormone regulation of proopiomelanocortin gene expression in arcuate neurons that innervate the medial preoptic area of the rat. Neuroendocrinology.

[222] Cheung S, Salinas J, Hammer RP (1995). Gonadal steroid hormone-dependence of beta-endorphin-like immunoreactivity in the medial preoptic area of the rat. Brain Res.

[223] Horvath TL, Kalra SP, Naftolin F, Leranth C (1995). Morphological evidence for a galanin-opiate interaction in the rat mediobasal hypothalamus. J Neuroendocrinol.

[224] Allen DL, Johnson AE, Tempel A, Zukin RS, Luine VN, McEwen BS (1993). Serotonergic lesions decrease mu- and delta-opiate receptor binding in discrete areas of the hypothalamus and in the midbrain central gray. Brain Res.

[225] Torii M, Kubo K (1994). The effects of intraventricular injection of beta-endorphin on initial estrogen action to induce lordosis behavior. Physiol Behav.

[226] Gorzalka BB, Heddema GM, Lester GL, Hanson LA (1997). beta-endorphin inhibits and facilitates lordosis behaviour in rats depending on ventricular site of administration. Neuropeptides.

[227] Charbogne P, Kieffer BL, Befort K (2014). 15 years of genetic approaches in vivo for addiction research: Opioid receptor and peptide gene knockout in mouse models of drug abuse. Neuropharmacology.

[228] Roth-Deri I, Green-Sadan T, Yadid G (2008). Beta-endorphin and drug-induced reward and reinforcement. Prog Neurobiol.

[229] Monteleone P (2011). New frontiers in endocrinology of eating disorders. Curr Top Behav Neurosci.

[230] Pecina S, Smith KS (2010). Hedonic and motivational roles of opioids in food reward: implications for overeating disorders. Pharmacol Biochem Behav.

[231] Kringelbach ML, Berridge KC (2010). The functional neuroanatomy of pleasure and happiness. Discov Med.

[232] Kringelbach ML, Berridge KC (2010). The neuroscience of happiness and pleasure. Soc Res (New York).

[233] Berridge KC, Ho CY, Richard JM, DiFeliceantonio AG (2010). The tempted brain eats: pleasure and desire circuits in obesity and eating disorders. Brain Res.

[234] Berridge KC (2009). ‘Liking’ and ‘wanting’ food rewards: brain substrates and roles in eating disorders. Physiol Behav.

[235] Benton D (2010). The plausibility of sugar addiction and its role in obesity and eating disorders. Clin Nutr.

[236] Fulton S (2010). Appetite and reward. Front Neuroendocrinol.

[237] Gosnell BA, Levine AS (2009). Reward systems and food intake: role of opioids. Int J Obes (Lond).

[238] Mahler SV, Berridge KC (2009). Which cue to “want?” Central amygdala opioid activation enhances and focuses incentive salience on a prepotent reward cue. J Neurosci.

[239] Nathan PJ, Bullmore ET (2009). From taste hedonics to motivational drive: central mu-opioid receptors and binge-eating behaviour. Int J Neuropsychopharmacol.

[240] Reece AS (2011). Hypothalamic opioid-melanocortin appetitive balance and addictive craving. Med Hypotheses.

[241] Yamamoto T (2008). Central mechanisms of roles of taste in reward and eating. Acta Physiol Hung.

[242] Cox LA, Nathanielsz PW (2009). The importance of altered gene promoter methylation and transcription factor binding in developmental programming of central appetitive drive. J Physiol.

[243] Hasan TF, Hasan H (2011). Anorexia nervosa: a unified neurological perspective. Int J Med Sci.

[244] Bandelow B, Schmahl C, Falkai P, Wedekind D (2010). Borderline personality disorder: a dysregulation of the endogenous opioid system?. Psychol Rev.

[245] Camacho FJ, Portillo W, Quintero-Enriquez O, Paredes RG (2009). Reward value of intromissions and morphine in male rats evaluated by conditioned place preference. Physiol Behav.

[246] Kippin TE, van der Kooy D (2003). Excitotoxic lesions of the tegmental pedunculopontine nucleus impair copulation in naive male rats and block the rewarding effects of copulation in experienced male rats. Eur J Neurosci.

[247] Nocjar C, Panksepp J (2007). Prior morphine experience induces long-term increases in social interest and in appetitive behavior for natural reward. Behav Brain Res.

[248] Olivier JD, de Jong TR, Jos Dederen P, van Oorschot R, Heeren D, Pattij T (2007). Effects of acute and chronic apomorphine on sex behavior and copulation-induced neural activation in the male rat. Eur J Pharmacol.

[249] Paredes RG (2009). Evaluating the neurobiology of sexual reward. ILAR J.

[250] Pfaus JG (2009). Pathways of sexual desire. J Sex Med.

[251] Pitchers KK, Frohmader KS, Vialou V, Mouzon E, Nestler EJ, Lehman MN (2010). DeltaFosB in the nucleus accumbens is critical for reinforcing effects of sexual reward. Genes Brain Behav.

[252] Riters LV (2010). Evidence for opioid involvement in the motivation to sing. J Chem Neuroanat.

[253] Tenk CM, Wilson H, Zhang Q, Pitchers KK, Coolen LM (2009). Sexual reward in male rats: effects of sexual experience on conditioned place preferences associated with ejaculation and intromissions. Horm Behav.

[254] Pitchers KK, Balfour ME, Lehman MN, Richtand NM, Yu L, Coolen LM (2010). Neuroplasticity in the mesolimbic system induced by natural reward and subsequent reward abstinence. Biol Psychiatry.

[255] Camacho FJ, Garcia-Horsman P, Paredes RG (2009). Hormonal and testing conditions for the induction of conditioned place preference by paced mating. Horm Behav.

[256] Nguyen AT, Marquez P, Hamid A, Kieffer B, Friedman TC, Lutfy K (2012). The rewarding action of acute cocaine is reduced in beta-endorphin deficient but not in mu opioid receptor knockout mice. Eur J Pharmacol.

[257] Simmons D, Self DW (2009). Role of mu- and delta-opioid receptors in the nucleus accumbens in cocaine-seeking behavior. Neuropsychopharmacology.

[258] Tseng A, Nguyen K, Hamid A, Garg M, Marquez P, Lutfy K (2013). The role of endogenous beta-endorphin and enkephalins in ethanol reward. Neuropharmacology.

[259] Zellner MR, Watt DF, Solms M, Panksepp J (2011). Affective neuroscientific and neuropsychoanalytic approaches to two intractable psychiatric problems: why depression feels so bad and what addicts really want. Neurosci Biobehav Rev.

[260] Niikura K, Narita M, Butelman ER, Kreek MJ, Suzuki T (2010). Neuropathic and chronic pain stimuli downregulate central mu-opioid and dopaminergic transmission. Trends Pharmacol Sci.

[261] Trezza V, Damsteegt R, Achterberg EJ, Vanderschuren LJ (2011). Nucleus accumbens mu-opioid receptors mediate social reward. J Neurosci.

[262] Nestler EJ (2005). The neurobiology of cocaine addiction. Sci Pract Perspect.

[263] Nestler EJ (2005). Is there a common molecular pathway for addiction?. Nat Neurosci.

[264] Di Chiara G, Imperato A (1988). Drugs abused by humans preferentially increase synaptic dopamine concentrations in the mesolimbic system of freely moving rats. Proc Natl Acad Sci U S A.

[265] Spanagel R, Herz A, Shippenberg TS (1990). Identification of the opioid receptor types mediating beta-endorphin-induced alterations in dopamine release in the nucleus accumbens. Eur J Pharmacol.

[266] Spanagel R, Herz A, Shippenberg TS (1990). The effects of opioid peptides on dopamine release in the nucleus accumbens: an in vivo microdialysis study. J Neurochem.

[267] Spanagel R, Weiss F (1999). The dopamine hypothesis of reward: past and current status. Trends Neurosci.

[268] Hadjiconstantinou M, Neff NH (2011). Nicotine and endogenous opioids: neurochemical and pharmacological evidence. Neuropharmacology.

[269] Charmandari E, Tsigos C, Chrousos G (2005). Endocrinology of the stress response. Annu Rev Physiol.

[270] Sarnyai Z, Shaham Y, Heinrichs SC (2001). The role of corticotropin-releasing factor in drug addiction. Pharmacol Rev.

[271] Sauriyal DS, Jaggi AS, Singh N (2011). Extending pharmacological spectrum of opioids beyond analgesia: multifunctional aspects in different pathophysiological states. Neuropeptides.

[272] Tsigos C, Chrousos GP (2002). Hypothalamic-pituitary-adrenal axis, neuroendocrine factors and stress. J Psychosom Res.

[273] Esch T, Stefano GB (2010). The neurobiology of stress management. Neuro Endocrinol Lett.

[274] Goeders NE, Guerin GF (1994). Non-contingent electric footshock facilitates the acquisition of intravenous cocaine self-administration in rats. Psychopharmacology (Berl).

[275] Bilavsky E, Dagan A, Yarden-Bilavsky H, Davidovits M, Shapiro R, Mor E (2011). Adrenal insufficiency during physiological stress in children after kidney or liver transplantation. Pediatr Transplant.

[276] Green-Sadan T, Kinor N, Roth-Deri I, Geffen-Aricha R, Schindler CJ, Yadid G (2003). Transplantation of glial cell line-derived neurotrophic factor-expressing cells into the striatum and nucleus accumbens attenuates acquisition of cocaine self-administration in rats. Eur J Neurosci.

[277] Hadjiconstantinou M, Duchemin AM, Zhang H, Neff NH (2011). Enhanced dopamine transporter function in striatum during nicotine withdrawal. Synapse.

[278] Shi J, Li SX, Zhang XL, Wang X, Le Foll B, Zhang XY (2009). Time-dependent neuroendocrine alterations and drug craving during the first month of abstinence in heroin addicts. Am J Drug Alcohol Abuse.

[279] Bachtell RK, Self DW (2009). Effects of adenosine A2A receptor stimulation on cocaine-seeking behavior in rats. Psychopharmacology (Berl).

[280] Ahmed SH, Koob GF (2005). Transition to drug addiction: a negative reinforcement model based on an allostatic decrease in reward function. Psychopharmacology (Berl).

[281] Barfield ET, Barry SM, Hodgin HB, Thompson BM, Allen SS, Grisel JE (2010). Beta-endorphin mediates behavioral despair and the effect of ethanol on the tail suspension test in mice. Alcohol Clin Exp Res.

[282] Sapolsky RM (2004). Why zebra's don’t get ulcers.

[283] Swanson LW, Sawchenko PE (1983). Hypothalamic integration: organization of the paraventricular and supraoptic nuclei. Annu Rev Neurosci.

[284] Swanson LW, Sawchenko PE, Lind RW, Rho JH (1987). The CRH motoneuron: differential peptide regulation in neurons with possible synaptic, paracrine, and endocrine outputs. Ann N Y Acad Sci.

[285] Swanson LW, Björklund A, Hökfelt T, Swanson LW (1987). The hypothalamus. Integrated systems of the CNS, part 1.

[286] Veening JG, van der Meer MJ, Joosten H, Hermus AR, Rijnnkels CE, Geeraedts LM (1993). Intravenous administration of interleukin-1 beta induces Fos-like immunoreactivity in corticotropin-releasing hormone neurons in the paraventricular hypothalamic nucleus of the rat. J Chem Neuroanat.

[287] Veening JG, Coolen LM, Spooren WJ, Joosten H, van Oorschot R, Mos J (1998). Patterns of c-fos expression induced by fluvoxamine are different after acute vs. chronic oral administration. Eur Neuropsychopharmacol.

[288] Veening JG, Bouwknecht JA, Joosten HJ, Dederen PJ, Zethof TJ, Groenink L (2004). Stress-induced hyperthermia in the mouse: c-fos expression, corticosterone and temperature changes. Prog Neuropsychopharmacol Biol Psychiatry.

[289] Groenink L, Dirks A, Verdouw PM, Schipholt M, Veening JG, van der Gugten J (2002). HPA axis dysregulation in mice overexpressing corticotropin releasing hormone. Biol Psychiatry.

[290] Veening JG, Bocker KB, Verdouw PM, Olivier B, De Jongh R, Groenink L (2009). Activation of the septohippocampal system differentiates anxiety from fear in startle paradigms. Neuroscience.

[291] Grisel JE, Bartels JL, Allen SA, Turgeon VL (2008). Influence of beta-Endorphin on anxious behavior in mice: interaction with EtOH. Psychopharmacology (Berl).

[292] Bergasa NV, Rothman RB, Mukerjee E, Vergalla J, Jones EA (2002). Up-regulation of central mu-opioid receptors in a model of hepatic encephalopathy: a potential mechanism for increased sensitivity to morphine in liver failure. Life Sci.

[293] Quang PN, Schmidt BL (2010). Peripheral endothelin B receptor agonist-induced antinociception involves endogenous opioids in mice. Pain.

[294] Hagerty MR, Isaacs J, Brasington L, Shupe L, Fetz EE, Cramer SC (2013). Case study of ecstatic meditation: fMRI and EEG evidence of self-stimulating a reward system. Neural Plast.

[295] Harte JL, Eifert GH (1995). The effects of running, environment, and attentional focus on athletes’ catecholamine and cortisol levels and mood. Psychophysiology.

[296] Harte JL, Eifert GH, Smith R (1995). The effects of running and meditation on beta-endorphin, corticotropin-releasing hormone and cortisol in plasma, and on mood. Biol Psychol.

[297] Infante JR, Peran F, Martinez M, Roldan A, Poyatos R, Ruiz C (1998). ACTH and beta-endorphin in transcendental meditation. Physiol Behav.

[298] Koob GF, Le Moal M (2005). Plasticity of reward neurocircuitry and the ‘dark side’ of drug addiction. Nat Neurosci.

[299] Papathanassoglou ED, Giannakopoulou M, Mpouzika M, Bozas E, Karabinis A (2010). Potential effects of stress in critical illness through the role of stress neuropeptides. Nurs Crit Care.

[300] Roth-Deri I, Zangen A, Aleli M, Goelman RG, Pelled G, Nakash R (2003). Effect of experimenter-delivered and self-administered cocaine on extracellular beta-endorphin levels in the nucleus accumbens. J Neurochem.

[301] Veehof MM, Oskam MJ, Schreurs KM, Bohlmeijer ET (2011). Acceptance-based interventions for the treatment of chronic pain: a systematic review and meta-analysis. Pain.

[302] Yadav RK, Magan D, Mehta M, Mehta N, Mahapatra SC (2012). A short-term, comprehensive, yoga-based lifestyle intervention is efficacious in reducing anxiety, improving subjective well-being and personality. Int J Yoga.

[303] Slominski A, Wortsman J, Luger T, Paus R, Solomon S (2000). Corticotropin releasing hormone and proopiomelanocortin involvement in the cutaneous response to stress. Physiol Rev.

[304] Tsigos C, Crosby SR, Gibson S, Young RJ, White A (1993). Proopiomelanocortin is the predominant adrenocorticotropin-related peptide in human cerebrospinal fluid. J Clin Endocrinol Metab.

[305] de Kloet ER (2009). Stress: a neurobiological perspective. Tijdschr Psychiatr.

[306] de Kloet ER, Fitzsimons CP, Datson NA, Meijer OC, Vreugdenhil E (2009). Glucocorticoid signaling and stress-related limbic susceptibility pathway: about receptors, transcription machinery and microRNA. Brain Res.

[307] Yadav RK, Magan D, Mehta N, Sharma R, Mahapatra SC (2012). Efficacy of a short-term yoga-based lifestyle intervention in reducing stress and inflammation: preliminary results. J Altern Complement Med.

[308] de Kloet ER (2010). From vasotocin to stress and cognition. Eur J Pharmacol.

[309] Shapiro SL, Brown KW, Thoresen C, Plante TG (2011). The moderation of Mindfulness-based stress reduction effects by trait mindfulness: results from a randomized controlled trial. J Clin Psychol.

[310] Dobkin PL, Zhao Q (2011). Increased mindfulness–the active component of the mindfulness-based stress reduction program?. Complement Ther Clin Pract.

[311] Schmidt S, Grossman P, Schwarzer B, Jena S, Naumann J, Walach H (2011). Treating fibromyalgia with mindfulness-based stress reduction: results from a 3-armed randomized controlled trial. Pain.

[312] Esmer G, Blum J, Rulf J, Pier J (2010). Mindfulness-based stress reduction for failed back surgery syndrome: a randomized controlled trial. J Am Osteopath Assoc.

[313] Merkes M (2010). Mindfulness-based stress reduction for people with chronic diseases. Aust J Prim Health.

[314] Goldin PR, Gross JJ (2010). Effects of mindfulness-based stress reduction (MBSR) on emotion regulation in social anxiety disorder. Emotion.

[315] Perlman DM, Salomons TV, Davidson RJ, Lutz A (2010). Differential effects on pain intensity and unpleasantness of two meditation practices. Emotion.

[316] Rosenzweig S, Greeson JM, Reibel DK, Green JS, Jasser SA, Beasley D (2010). Mindfulness-based stress reduction for chronic pain conditions: variation in treatment outcomes and role of home meditation practice. J Psychosom Res.

[317] Chiesa A (2010). Vipassana meditation: systematic review of current evidence. J Altern Complement Med.

[318] Chiesa A, Calati R, Serretti A (2011). Does mindfulness training improve cognitive abilities? A systematic review of neuropsychological findings. Clin Psychol Rev.

[319] Chiesa A, Serretti A (2011). Mindfulness based cognitive therapy for psychiatric disorders: a systematic review and meta-analysis. Psychiatry Res.

[320] Zeidan F, Martucci KT, Kraft RA, Gordon NS, McHaffie JG, Coghill RC (2011). Brain mechanisms supporting the modulation of pain by mindfulness meditation. J Neurosci.

[321] Abbott RA, Whear R, Rodgers LR, Bethel A, Thompson Coon J, Kuyken W (2014). Effectiveness of mindfulness-based stress reduction and mindfulness based cognitive therapy in vascular disease: a systematic review and meta-analysis of randomised controlled trials. J Psychosom Res.

[322] Black DS (2014). Mindfulness-based interventions: an antidote to suffering in the context of substance use, misuse, and addiction. Subst Use Misuse.

[323] Eisendrath SJ, Gillung EP, Delucchi KL, Chartier M, Mathalon DH, Sullivan JC (2014). Mindfulness-based cognitive therapy (MBCT) versus the health-enhancement program (HEP) for adults with treatment-resistant depression: a randomized control trial study protocol. BMC Complement Altern Med.

[324] Kopf S, Oikonomou D, Hartmann M, Feier F, Faude-Lang V, Morcos M (2014). Effects of stress reduction on cardiovascular risk factors in type 2 diabetes patients with early kidney disease - results of a randomized controlled trial (HEIDIS). Exp Clin Endocrinol Diabetes.

[325] Lenze EJ, Hickman S, Hershey T, Wendleton L, Ly K, Dixon D (2014). Mindfulness-based stress reduction for older adults with worry symptoms and co-occurring cognitive dysfunction. Int J Geriatr Psychiatry.

[326] Smith SA (2014). Mindfulness-Based Stress Reduction: An Intervention to Enhance the Effectiveness of Nurses' Coping With Work-Related Stress. Int J Nurs Knowl.

[327] Rapgay L, Bystrisky A (2009). Classical mindfulness: an introduction to its theory and practice for clinical application. Ann N Y Acad Sci.

[328] Chiesa A, Serretti A (2010). A systematic review of neurobiological and clinical features of mindfulness meditations. Psychol Med.

[329] Moynihan JA, Chapman BP, Klorman R, Krasner MS, Duberstein PR, Brown KW (2013). Mindfulness-based stress reduction for older adults: effects on executive function, frontal alpha asymmetry and immune function. Neuropsychobiology.

[330] Pickut BA, Van Hecke W, Kerckhofs E, Marien P, Vanneste S, Cras P (2013). Mindfulness based intervention in Parkinson’s disease leads to structural brain changes on MRI: a randomized controlled longitudinal trial. Clin Neurol Neurosurg.

[331] Wells RE, Yeh GY, Kerr CE, Wolkin J, Davis RB, Tan Y (2013). Meditation’s impact on default mode network and hippocampus in mild cognitive impairment: a pilot study. Neurosci Lett.

[332] Holzel BK, Carmody J, Vangel M, Congleton C, Yerramsetti SM, Gard T (2011). Mindfulness practice leads to increases in regional brain gray matter density. Psychiatry Res.

[333] Holzel BK, Carmody J, Evans KC, Hoge EA, Dusek JA, Morgan L (2010). Stress reduction correlates with structural changes in the amygdala. Soc Cogn Affect Neurosci.

[334] Jovanovic H, Perski A, Berglund H, Savic I (2011). Chronic stress is linked to 5-HT(1A) receptor changes and functional disintegration of the limbic networks. Neuroimage.

[335] Kerr CE, Sacchet MD, Lazar SW, Moore CI, Jones SR (2013). Mindfulness starts with the body: somatosensory attention and top-down modulation of cortical alpha rhythms in mindfulness meditation. Front Hum Neurosci.

[336] Goldin P, Ziv M, Jazaieri H, Hahn K, Gross JJ (2013). MBSR vs aerobic exercise in social anxiety: fMRI of emotion regulation of negative self-beliefs. Soc Cogn Affect Neurosci.

[337] Hegadoren KM, O’Donnell T, Lanius R, Coupland NJ, Lacaze-Masmonteil N (2009). The role of beta-endorphin in the pathophysiology of major depression. Neuropeptides.

[338] Panksepp J (2010). Affective neuroscience of the emotional BrainMind: evolutionary perspectives and implications for understanding depression. Dialogues Clin Neurosci.

[339] Coenen VA, Schlaepfer TE, Maedler B, Panksepp J (2011). Cross-species affective functions of the medial forebrain bundle-implications for the treatment of affective pain and depression in humans. Neurosci Biobehav Rev.

[340] Harro J, Kanarik M, Matrov D, Panksepp J (2011). Mapping patterns of depression-related brain regions with cytochrome oxidase histochemistry: relevance of animal affective systems to human disorders, with a focus on resilience to adverse events. Neurosci Biobehav Rev.

[341] Bernroider G, Panksepp J (2011). Mirrors and feelings: have you seen the actors outside?. Neurosci Biobehav Rev.

[342] Panksepp J (2011). Toward a cross-species neuroscientific understanding of the affective mind: do animals have emotional feelings?. Am J Primatol.

[343] Stein DJ, van Honk J, Ipser J, Solms M, Panksepp J (2007). Opioids: from physical pain to the pain of social isolation. CNS Spectr.

[344] Panksepp J, Herman BH, Vilberg T, Bishop P, DeEskinazi FG (1980). Endogenous opioids and social behavior. Neurosci Biobehav Rev.

[345] Akil H, Richardson DE, Barchas JD, Li CH (1978). Appearance of beta-endorphin-like immunoreactivity in human ventricular cerebrospinal fluid upon analgesic electrical stimulation. Proc Natl Acad Sci U S A.

[346] Akil H, Richardson DE, Hughes J, Barchas JD (1978). Enkephalin-like material elevated in ventricular cerebrospinal fluid of pain patients after analgetic focal stimulation. Science.

[347] Guillemin R, Ling N, Burgus R, Bloom F, Segal D (1977). Characterization of the endorphins, novel hypothalamic and neurohypophysial peptides with opiate-like activity: evidence that they induce profound behavioral changes. Psychoneuroendocrinology.

[348] Guillemin R, Ling N, Lazarus L, Burgus R, Minick S, Bloom F (1977). The endorphins, novel peptides of brain and hypophysial origin, with opiate-like activity: biochemical and biologic studies. Ann N Y Acad Sci.

[349] Segal DS, Browne RG, Bloom F, Ling N, Guillemin R (1977). beta-Endorphin: endogenous opiate or neuroleptic?. Science.

[350] Bloom F, Battenberg E, Rossier J, Ling N, Leppaluoto J, Vargo TM (1977). Endorphins are located in the intermediate and anterior lobes of the pituitary gland, not in the neurohypophysis. Life Sci.

[351] Bloom F, Segal D, Ling N, Guillemin R (1976). Endorphins: profound behavioral effects in rats suggest new etiological factors in mental illness. Science.

[352] Frederickson RC, Geary LE (1982). Endogenous opioid peptides: review of physiological, pharmacological and clinical aspects. Prog Neurobiol.

[353] O’Donohue TL, Dorsa DM (1982). The opiomelanotropinergic neuronal and endocrine systems. Peptides.

[354] Loh HH, Tseng LF, Wei E, Li CH (1976). beta-endorphin is a potent analgesic agent. Proc Natl Acad Sci U S A.

[355] Foley KM, Kourides IA, Inturrisi CE, Kaiko RF, Zaroulis CG, Posner JB (1979). beta-Endorphin: analgesic and hormonal effects in humans. Proc Natl Acad Sci U S A.

[356] Backryd E, Ghafouri B, Larsson B, Gerdle B (2014). Do low levels of beta-endorphin in the cerebrospinal fluid indicate defective top-down inhibition in patients with chronic neuropathic pain? A cross-sectional, comparative study. Pain Med.

[357] Feldreich A, Ernberg M, Lund B, Rosen A (2012). Increased beta-endorphin levels and generalized decreased pain thresholds in patients with limited jaw opening and movement-evoked pain from the temporomandibular joint. J Oral Maxillofac Surg.

[358] Matejec R, Ruwoldt R, Bodeker RH, Hempelmann G, Teschemacher H (2003). Release of beta-endorphin immunoreactive material under perioperative conditions into blood or cerebrospinal fluid: significance for postoperative pain?. Anesth Analg.

[359] Morales AB, Vives F, Ros I, Mora F (1988). Plasma and CSF levels of immunoreactive beta-endorphin in algic peaks of patients with herniated intervertebral discs. Rev Esp Fisiol.

[360] Salar G, Mingrino S, Trabucchi M, Bosio A, Semenza C (1981). Evaluation of endorphin content in the CSF of patients with trigeminal neuralgia before and after Gasserian ganglion thermocoagulation. J Neurosurg.

[361] Imasato H, Nagata K, Hashimoto S, Komori H, Inoue A (1997). Objective evaluation of pain in various spinal diseases: neuropeptide immunoreactivity in the cerebrospinal fluid. Spinal Cord.

[362] Tsigos C, Gibson S, Crosby SR, White A, Young RJ (1995). Cerebrospinal fluid levels of beta endorphin in painful and painless diabetic polyneuropathy. J Diabetes Complications.

[363] Vaeroy H, Helle R, Forre O, Kass E, Terenius L (1988). Cerebrospinal fluid levels of beta-endorphin in patients with fibromyalgia (fibrositis syndrome). J Rheumatol.

[364] Vaeroy H, Nyberg F, Terenius L (1991). No evidence for endorphin deficiency in fibromyalgia following investigation of cerebrospinal fluid (CSF) dynorphin A and Met-enkephalin-Arg6-Phe7. Pain.

[365] Nappi G, Facchinetti F, Martignoni E, Petraglia F, Bono G, Micieli G (1985). Plasma and CSF endorphin levels in primary and symptomatic headaches. Headache.

[366] Jorgensen LS, Bach FW, Christiansen P, Raundahl U, Ostgaard S, Ekman R (1993). Decreased cerebrospinal fluid beta-endorphin and increased pain sensitivity in patients with functional abdominal pain. Scand J Gastroenterol.

[367] Jorgensen LS, Bach FW, Christiansen PM, Raundahl U, Ostgaard SE, Ekman R (1995). Reduced concentration of beta-endorphin in cerebrospinal fluid and reduced pain tolerance in patients with functional dyspepsia. Ugeskr Laeger.

[368] Genazzani AR, Nappi G, Facchinetti F, Micieli G, Petraglia F, Bono G (1984). Progressive impairment of CSF beta-EP levels in migraine sufferers. Pain.

[369] Misra UK, Kalita J, Tripathi GM, Bhoi SK (2013). Is beta endorphin related to migraine headache and its relief?. Cephalalgia.

[370] Wu XF, Zhang MK, Huang H (2014). Evaluation of analgesic, sedative effects and antimigraine mechanism of Qilong Toutong Granule () in rodents. Chin J Integr Med.

[371] Zhao Y, Tian L, Sheng W, Miao J, Yang J (2013). Hypalgesia effect of IL-24, a quite new mechanism for IL-24 application in cancer treatment. J Interferon Cytokine Res.

[372] Facchinetti F, Petraglia F, Nappi G, Martignoni E, Sinforiani E, Bono G (1984). Functional opioid activity variates according to the different fashion of alcohol abuse. Subst Alcohol Actions Misuse.

[373] Bach FW (1997). Beta-endorphin in the brain. A role in nociception. Acta Anaesthesiol Scand.

[374] Bach FW, Chaplan SR, Jang J, Yaksh TL (1995). Cerebrospinal fluid beta-endorphin in models of hyperalgesia in the rat. Regul Pept.

[375] Bach FW, Yaksh TL (1995). Release of beta-endorphin immunoreactivity into ventriculo-cisternal perfusate by lumbar intrathecal capsaicin in the rat. Brain Res.

[376] Misra UK, Kalita J, Bhoi SK (2013). High-rate repetitive transcranial magnetic stimulation in migraine prophylaxis: a randomized, placebo-controlled study. J Neurol.

[377] Young RF, Bach FW, Van Norman AS, Yaksh TL (1993). Release of beta-endorphin and methionine-enkephalin into cerebrospinal fluid during deep brain stimulation for chronic pain. Effects of stimulation locus and site of sampling. J Neurosurg.

[378] Hosobuchi Y (1981). Periaqueductal gray stimulation in humans produces analgesia accompanied by elevation of beta-endorphin and ACTH in ventricular CSF. Mod Probl Pharmacopsychiatry.

[379] Shen J (2001). Research on the neurophysiological mechanisms of acupuncture: review of selected studies and methodological issues. J Altern Complement Med.

[380] Zhao ZQ (2008). Neural mechanism underlying acupuncture analgesia. Prog Neurobiol.

[381] Lee HJ, Lee JH, Lee EO, Lee HJ, Kim KH, Lee KS (2009). Substance P and beta endorphin mediate electroacupuncture induced analgesic activity in mouse cancer pain model. Acupunct Electrother Res.

[382] Zyloney CE, Jensen K, Polich G, Loiotile RE, Cheetham A, LaViolette PS (2010). Imaging the functional connectivity of the Periaqueductal Gray during genuine and sham electroacupuncture treatment. Mol Pain.

[383] He LF, Dong WQ (1983). Activity of opioid peptidergic system in acupuncture analgesia. Acupunct Electrother Res.

[384] Ho WK, Wen HL (1989). Opioid-like activity in the cerebrospinal fluid of pain patients treated by electroacupuncture. Neuropharmacology.

[385] Guo ZL, Longhurst JC (2010). Activation of reciprocal pathways between arcuate nucleus and ventrolateral periaqueductal gray during electroacupuncture: involvement of VGLUT3. Brain Res.

[386] Li P, Tjen ALSC, Guo ZL, Longhurst JC (2010). An arcuate-ventrolateral periaqueductal gray reciprocal circuit participates in electroacupuncture cardiovascular inhibition. Auton Neurosci.

[387] Guo J, Liu J, Fu W, Ma W, Xu Z, Yuan M (2008). Effect of electroacupuncture stimulation of hindlimb on seizure incidence and supragranular mossy fiber sprouting in a rat model of epilepsy. J Physiol Sci.

[388] Guo J, Liu J, Fu W, Ma W, Xu Z, Yuan M (2008). The effect of electroacupuncture on spontaneous recurrent seizure and expression of GAD(67) mRNA in dentate gyrus in a rat model of epilepsy. Brain Res.

[389] Cao X (2002). Scientific bases of acupuncture analgesia. Acupunct Electrother Res.

[390] Cheng LL, Ding MX, Xiong C, Zhou MY, Qiu ZY, Wang Q (2012). Effects of electroacupuncture of different frequencies on the release profile of endogenous opioid peptides in the central nerve system of goats. Evid Based Complement Alternat Med.

[391] Nakamura T, Tomida M, Yamamoto T, Ando H, Takamata T, Kondo E (2013). The endogenous opioids related with antinociceptive effects induced by electrical stimulation into the amygdala. Open Dent J.

[392] Ren XX, Guo MW, Zhao YF, Ding XY, Li CH, Ji B (2012). Effects of electroacupuncture on pain reactions, expression of spinal kappa-opioid receptor and contents of enkephalin and beta-endorphin in periaqueductal gray of midbrain in dysmenorrhea model rats. Zhen Ci Yan Jiu.

[393] Wang Y, Gehringer R, Mousa SA, Hackel D, Brack A, Rittner HL (2014). CXCL10 Controls Inflammatory Pain via Opioid Peptide-Containing Macrophages in Electroacupuncture. PLoS One.

[394] Zhang Y, Zhang RX, Zhang M, Shen XY, Li A, Xin J (2012). Electroacupuncture inhibition of hyperalgesia in an inflammatory pain rat model: involvement of distinct spinal serotonin and norepinephrine receptor subtypes. Br J Anaesth.

[395] Chen P, Zhao BX, Qin W, Chen HY, Tian J, Fan YP (2008). Study on the mechanism of acupuncture at Daling (PC 7) for mental diseases by fMRI. Zhongguo Zhen Jiu.

[396] Alessi NE, Khachaturian H, Watson S, Akil H (1983). Postnatal ontogeny of acetylated and non-acetylated B-endorphin in rat pituitary. Life Sci.

[397] Banks WA, Kastin AJ (1989). Quantifying carrier-mediated transport of peptides from the brain to the blood. Methods Enzymol.

[398] Scheef L, Jankowski J, Daamen M, Weyer G, Klingenberg M, Renner J (2012). An fMRI study on the acute effects of exercise on pain processing in trained athletes. Pain.

[399] Bender T, Nagy G, Barna I, Tefner I, Kadas E, Geher P (2007). The effect of physical therapy on beta-endorphin levels. Eur J Appl Physiol.

[400] Spaziante R, Merola B, Colao A, Gargiulo G, Cafiero T, Irace C (1990). Beta-endorphin concentrations both in plasma and in cerebrospinal fluid in response to acute painful stimuli. J Neurosurg Sci.

[401] Zangen A, Nakash R, Yadid G (1999). Serotonin-mediated increases in the extracellular levels of beta-endorphin in the arcuate nucleus and nucleus accumbens: a microdialysis study. J Neurochem.

[402] Basbaum AI, Fields HL (1984). Endogenous pain control systems: brainstem spinal pathways and endorphin circuitry. Annu Rev Neurosci.

[403] Fields H (2004). State-dependent opioid control of pain. Nat Rev Neurosci.

[404] Zelinski LM, Ohgami Y, Quock RM (2009). Exposure to nitrous oxide stimulates a nitric oxide-dependent neuronal release of beta-endorphin in ventricular-cisternally-perfused rats. Brain Res.

[405] Zubrzycka M, Janecka A (2011). Effect of tooth pulp and periaqueductal central gray electrical stimulation on beta-endorphin release into the fluid perfusing the cerebral ventricles in rats. Brain Res.

[406] Guo HF, Tian J, Wang X, Fang Y, Hou Y, Han J (1996). Brain substrates activated by electroacupuncture of different frequencies (I): Comparative study on the expression of oncogene c-fos and genes coding for three opioid peptides. Brain Res Mol Brain Res.

[407] Guo X, Zhao H, Wang Y, Su W (2008). Advances of clinical studies on acupuncture treatment of depression. Zhen Ci Yan Jiu.

[408] Spetea M (2013). Opioid receptors and their ligands in the musculoskeletal system and relevance for pain control. Curr Pharm Des.

[409] Akil H, Watson SJ, Sullivan S, Barchas JD (1978). Enkephalin-like material in normal human CSF: measurement and levels. Life Sci.

[410] Bach FW, Yaksh TL (1995). Release of beta-endorphin immunoreactivity from brain by activation of a hypothalamic N-methyl-D-aspartate receptor. Neuroscience.

[411] Yadid G, Zangen A, Herzberg U, Nakash R, Sagen J (2000). Alterations in endogenous brain beta-endorphin release by adrenal medullary transplants in the spinal cord. Neuropsychopharmacology.

[412] Zubrzycka M, Szemraj J, Janecka A (2011). Effect of tooth pulp and periaqueductal central gray stimulation on the expression of genes encoding the selected neuropeptides and opioid receptors in the mesencephalon, hypothalamus and thalamus in rats. Brain Res.

[413] Baumgarten CR, Schmitz P, O’Connor A, Kunkel G (2002). Effects of beta-endorphin on nasal allergic inflammation. Clin Exp Allergy.

[414] Niu H, Zheng Y, Huma T, Rizak JD, Li L, Wang G (2013). Lesion of olfactory epithelium attenuates expression of morphine-induced behavioral sensitization and reinstatement of drug-primed conditioned place preference in mice. Pharmacol Biochem Behav.

[415] Jagannadha Rao A, Moudal NR, Li CH (1986). beta-Endorphin: intranasal administration increases the serum prolactin level in monkey. Int J Pept Protein Res.

[416] El-Sherif AE, Salem M, Yahia H, Al-Sharkawy WA, Al-Sayrafi M (1995). Treatment of renal colic by desmopressin intranasal spray and diclofenac sodium. J Urol.

[417] Franceschini R, Cataldi A, Barreca T, Salvemini M, Rolandi E (1989). Plasma beta-endorphin, ACTH and cortisol secretion in man after nasal spray administration of calcitonin. Eur J Clin Pharmacol.

[418] Vescovi PP, Pedrazzoni M, Gerra G, Maninetti L, Michelini M, Pioli G (1990). Salmon calcitonin given by nasal spray or by injection does not increase beta-endorphin levels in normal men. Life Sci.

[419] Westin U, Piras E, Jansson B, Bergstrom U, Dahlin M, Brittebo E (2005). Transfer of morphine along the olfactory pathway to the central nervous system after nasal administration to rodents. Eur J Pharm Sci.

[420] Westin UE, Bostrom E, Grasjo J, Hammarlund-Udenaes M, Bjork E (2006). Direct nose-to-brain transfer of morphine after nasal administration to rats. Pharm Res.

[421] Bulloch MN, Hutchison AM (2013). Fentanyl pectin nasal spray: a novel intranasal delivery method for the treatment of breakthrough cancer pain. Expert Rev Clin Pharmacol.

[422] Hitt JM, Corcoran T, Michienzi K, Creighton P, Heard C (2014). An evaluation of intranasal sufentanil and dexmedetomidine for pediatric dental sedation. Pharmaceutics.

[423] Leppert W (2013). New treatment possibilities for opioid-induced bowel dysfunction. Pain.

[424] Mercadante S, Prestia G, Adile C, Casuccio A (2014). Intranasal Fentanyl Versus Fentanyl Pectin Nasal Spray for the Management of Breakthrough Cancer Pain in Doses Proportional to Basal Opioid Regimen. J Pain.

[425] Nave R, Schmitt H, Popper L (2013). Faster absorption and higher systemic bioavailability of intranasal fentanyl spray compared to oral transmucosal fentanyl citrate in healthy subjects. Drug Deliv.

[426] Perelman M, Fisher AN, Smith A, Knight A (2013). Impact of allergic rhinitis and its treatment on the pharmacokinetics of nasally administered fentanyl. Int J Clin Pharmacol Ther.

[427] Bjelke B, Fuxe K (1993). Intraventricular beta-endorphin accumulates in DARPP-32 immunoreactive tanycytes. Neuroreport.

[428] Fuxe K, Li XM, Bjelke B, Hedlund PB, Biagini G, Agnati LF (1994). Possible mechanisms for the powerful actions of neuropeptides. Ann N Y Acad Sci.

[429] Bjelke B, Stromberg I, O’Connor WT, Andbjer B, Agnati LF, Fuxe K (1994). Evidence for volume transmission in the dopamine denervated neostriatum of the rat after a unilateral nigral 6-OHDA microinjection. Studies with systemic D-amphetamine treatment. Brain Res.

[430] Siegan JB, Sagen J (1997). Attenuation of formalin pain responses in the rat by adrenal medullary transplants in the spinal subarachnoid space. Pain.

[431] Sagen J, Wang H, Pappas GD (1990). Adrenal medullary implants in the rat spinal cord reduce nociception in a chronic pain model. Pain.

[432] Finegold AA, Mannes AJ, Iadarola MJ (1999). A paracrine paradigm for in vivo gene therapy in the central nervous system: treatment of chronic pain. Hum Gene Ther.

[433] Parolaro D, Crema G, Sala M, Santagostino A, Giagnoni G, Gori E (1986). Intestinal effect and analgesia: evidence for different involvement of opioid receptor subtypes in periaqueductal gray matter. Eur J Pharmacol.

[434] Hill C, Dunbar JC (2002). The effects of acute and chronic alpha melanocyte stimulating hormone (alphaMSH) on cardiovascular dynamics in conscious rats. Peptides.

[435] Sarkar DK, Zhang C, Murugan S, Dokur M, Boyadjieva NI, Ortiguela M (2011). Transplantation of beta-endorphin neurons into the hypothalamus promotes immune function and restricts the growth and metastasis of mammary carcinoma. Cancer Res.

[436] Borner C, Lanciotti S, Koch T, Hollt V, Kraus J (2013). mu opioid receptor agonist-selective regulation of interleukin-4 in T lymphocytes. J Neuroimmunol.

[437] Day YJ, Liou JT, Lee CM, Lin YC, Mao CC, Chou AH (2014). Lack of interleukin-17 leads to a modulated micro-environment and amelioration of mechanical hypersensitivity after peripheral nerve injury in mice. Pain.

[438] Dougherty PM, Pellis NR, Dafny N (1990). The brain and the immune system: an intact immune system is essential for the manifestation of withdrawal in opiate addicted rats. Neuroscience.

[439] Fioravanti A, Cantarini L, Guidelli GM, Galeazzi M (2011). Mechanisms of action of spa therapies in rheumatic diseases: what scientific evidence is there?. Rheumatol Int.

[440] Fioravanti A, Perpignano G, Tirri G, Cardinale G, Gianniti C, Lanza CE (2007). Effects of mud-bath treatment on fibromyalgia patients: a randomized clinical trial. Rheumatol Int.

[441] Furui T, Satoh K, Asano Y, Shimosawa S, Hasuo M, Yaksh TL (1984). Increase of beta-endorphin levels in cerebrospinal fluid but not in plasma in patients with cerebral infarction. J Neurosurg.

[442] Hostetler ED, Sanabria-Bohorquez S, Eng W, Joshi AD, Patel S, Gibson RE (2013). Evaluation of [(1)(8)F]MK-0911, a positron emission tomography (PET) tracer for opioid receptor-like 1 (ORL1), in rhesus monkey and human. Neuroimage.

[443] Jonsdottir IH, Hellstrand K, Thoren P, Hoffmann P (2000). Enhancement of natural immunity seen after voluntary exercise in rats. Role of central opioid receptors. Life Sci.

[444] Liou JT, Liu FC, Mao CC, Lai YS, Day YJ (2011). Inflammation confers dual effects on nociceptive processing in chronic neuropathic pain model. Anesthesiology.

[445] Liu H, Shen X, Tang H, Li J, Xiang T, Yu W (2013). Using microPET imaging in quantitative verification of the acupuncture effect in ischemia stroke treatment. Sci Rep.

[446] McVeigh JG, McGaughey H, Hall M, Kane P (2008). The effectiveness of hydrotherapy in the management of fibromyalgia syndrome: a systematic review. Rheumatol Int.

[447] Teschemacher H, Koch G, Scheffler H, Hildebrand A, Brantl V (1990). Opioid peptides. Immunological significance?. Ann N Y Acad Sci.

[448] Ye D, Bu H, Guo G, Shu B, Wang W, Guan X (2014). Activation of CXCL10/CXCR3 Signaling Attenuates Morphine Analgesia: Involvement of Gi Protein. J Mol Neurosci.

[449] Berger PA, Watson SJ, Akil H, Barchas JD (1986). Investigating opioid peptides in schizophrenia and depression. Res Publ Assoc Res Nerv Ment Dis.

[450] Berger PA, Watson SJ, Akil H, Elliott GR, Rubin RT, Pfefferbaum A (1980). beta-Endorphin and schizophrenia. Arch Gen Psychiatry.

[451] Melrose PA, Knigge KM (1989). Topography of oxytocin and vasopressin neurons in the forebrain of Equus caballus: further support of proposed evolutionary relationships for proopiomelanocortin, oxytocin and vasopressin neurons. Brain Behav Evol.

[452] Joseph SA, Pilcher WH, Knigge KM (1985). Anatomy of the corticotropin-releasing factor and opiomelanocortin systems of the brain. Fed Proc.

[453] Knigge KM, Joseph SA (1984). Anatomy of the opioid-systems of the brain. Can J Neurol Sci.

[454] Summy-Long JY, Miller DS, Rosella-Dampman LM, Hartman RD, Emmert SE (1984). A functional role for opioid peptides in the differential secretion of vasopressin and oxytocin. Brain Res.

[455] Bodnar RJ, Nilaver G, Wallace MM, Badillo-Martinez D, Zimmerman EA (1984). Pain threshold changes in rats following central injection of beta-endorphin, met-enkephalin, vasopressin or oxytocin antisera. Int J Neurosci.

[456] Bicknell RJ, Chapman C, Leng G (1985). Effects of opioid agonists and antagonists on oxytocin and vasopressin release in vitro. Neuroendocrinology.

[457] Bicknell RJ, Chapman C, Leng G (1985). Neurohypophysial opioids and oxytocin secretion: source of inhibitory opioids. Exp Brain Res.

[458] Kovacs GL, Telegdy G (1987). Beta-endorphin tolerance is inhibited by oxytocin. Pharmacol Biochem Behav.

[459] Keverne EB (1988). Central mechanisms underlying the neural and neuroendocrine determinants of maternal behaviour. Psychoneuroendocrinology.

[460] Vecsernyes M, Laczi F, Kovacs GL, Szabo G, Janaky T, Telegdy G (1989). The effects of beta-endorphin on arginine-8-vasopressin and oxytocin levels in rat brain areas. Experientia.

[461] Csiffary A, Ruttner Z, Toth Z, Palkovits M (1992). Oxytocin nerve fibers innervate beta-endorphin neurons in the arcuate nucleus of the rat hypothalamus. Neuroendocrinology.

[462] Keverne EB, Kendrick KM (1994). Maternal behaviour in sheep and its neuroendocrine regulation. Acta Paediatr Suppl.

[463] Douglas AJ, Bicknell RJ, Russell JA (1995). Pathways to parturition. Adv Exp Med Biol.

[464] Douglas AJ, Leng G, Russell JA (2002). The importance of oxytocin mechanisms in the control of mouse parturition. Reproduction.

[465] Franchini LF, Rubinstein M, Vivas L (2003). Reduced sodium appetite and increased oxytocin gene expression in mutant mice lacking beta-endorphin. Neuroscience.

[466] Kutlu S, Yilmaz B, Canpolat S, Sandal S, Ozcan M, Kumru S (2004). Mu opioid modulation of oxytocin secretion in late pregnant and parturient rats. Involvement of noradrenergic neurotransmission. Neuroendocrinology.

[467] Bancroft J (2005). The endocrinology of sexual arousal. J Endocrinol.

[468] Yang J, Liu WY, Song CY, Lin BC (2006). Only arginine vasopressin, not oxytocin and endogenous opiate peptides, in hypothalamic paraventricular nucleus play a role in acupuncture analgesia in the rat. Brain Res Bull.

[469] Yang J, Yang Y, Xu HT, Chen JM, Liu WY, Lin BC (2007). Arginine vasopressin induces periaqueductal gray release of enkephalin and endorphin relating to pain modulation in the rat. Regul Pept.

[470] Vuong C, Van Uum SH, O’Dell LE, Lutfy K, Friedman TC (2010). The effects of opioids and opioid analogs on animal and human endocrine systems. Endocr Rev.

[471] Barna I, Sweep CG, Veldhuis HD, Wiegant VM, De Wied D (1990). Effects of pituitary beta-endorphin secretagogues on the concentration of beta-endorphin in rat cerebrospinal fluid: evidence for a role of vasopressin in the regulation of brain beta-endorphin release. Neuroendocrinology.

[472] Kjaer A, Larsen PJ, Knigge U, Moller M, Warberg J (1994). Histamine stimulates c-fos expression in hypothalamic vasopressin-, oxytocin-, and corticotropin-releasing hormone-containing neurons. Endocrinology.

[473] Morhenn V, Beavin LE, Zak PJ (2012). Massage increases oxytocin and reduces adrenocorticotropin hormone in humans. Altern Ther Health Med.

[474] Rosenblatt JS, Mayer AD, Giordano AL (1988). Hormonal basis during pregnancy for the onset of maternal behavior in the rat. Psychoneuroendocrinology.

[475] Sweep CG, Boomkamp MD, Barna I, Logtenberg AW, Wiegant VM (1990). Vasopressin enhances the clearance of beta-endorphin immunoreactivity from rat cerebrospinal fluid. Acta Endocrinol (Copenh).

[476] Yang J, Li P, Liang JY, Pan YJ, Yan XQ, Yan FL (2011). Oxytocin in the periaqueductal grey regulates nociception in the rat. Regul Pept.

[477] Yang J, Liang JY, Li P, Pan YJ, Qiu PY, Zhang J (2011). Oxytocin in the periaqueductal gray participates in pain modulation in the rat by influencing endogenous opiate peptides. Peptides.

[478] Yang J, Pan YJ, Zhao Y, Qiu PY, Lu L, Li P (2011). Oxytocin in the rat caudate nucleus influences pain modulation. Peptides.

[479] Douglas AJ, Bicknell RJ, Leng G, Russell JA, Meddle SL (2002). Beta-endorphin cells in the arcuate nucleus: projections to the supraoptic nucleus and changes in expression during pregnancy and parturition. J Neuroendocrinol.

[480] Sewards TV, Sewards MA (2003). Representations of motivational drives in mesial cortex, medial thalamus, hypothalamus and midbrain. Brain Res Bull.

[481] Sewards TV, Sewards MA (2003). Fear and power-dominance motivation: proposed contributions of peptide hormones present in cerebrospinal fluid and plasma. Neurosci Biobehav Rev.

